# Anti-Cancer Outcome of Glucocorticoid Receptor Transrepression by Synephrine Derivatives in Hematological Malignancies

**DOI:** 10.3390/ijms262311404

**Published:** 2025-11-25

**Authors:** Ekaterina M. Zhidkova, Ekaterina D. Savina, Daria V. Migaleva, Olga A. Vlasova, Timur T. Valiev, Adel D. Enikeev, Gennadii A. Badun, Maria G. Chernysheva, Svetlana A. Dodonova, Alexey A. Kryukov, Pavel A. Kusov, Kirill V. Gordeev, Ekaterina A. Yurchenko, Andrey V. Matveev, Marianna G. Yakubovskaya, Ekaterina A. Lesovaya

**Affiliations:** 1 Department of Chemical Carcinogenesis, Institute of Carcinogenesis, N.N. Blokhin National Medical Research Center of Oncology, Ministry of Health of Russia, Kashirskoe Shosse 24-15, Moscow 115478, Russiakaty.dm.savina@gmail.com (E.D.S.); darya.stepanycheva@yandex.ru (D.V.M.); olya_vlasov@mail.ru (O.A.V.); kusov.pavel@gmail.com (P.A.K.); mgyakubovskaya@mail.ru (M.G.Y.); 2 Department of Hemoblastosis Chemotherapy, Institute of Pediatric Oncology and Hematology, N.N. Blokhin National Medical Research Center of Oncology, Ministry of Health of Russia, Kashirskoe Shosse 24-15, Moscow 115478, Russia; timurvaliev@mail.ru; 3 Oncogene Regulation Department, Institute of Carcinogenesis, N.N. Blokhin National Medical Research Center of Oncology, Ministry of Health of Russia, Kashirskoe Shosse 24-15, Moscow 115478, Russia; adelbufyeni@mail.ru; 4 Department of Chemistry, M.V. Lomonosov Moscow State University, Leninskiye Gory 1, Moscow 119991, Russia; badunga@yandex.ru (G.A.B.); chernyshevamg@my.msu.ru (M.G.C.); 5 Department of Pathophysiology, Kursk State Medical University, Karl Marx St. 3, Kursk 305041, Russia; dodonovasveta@mail.ru (S.A.D.); kriukov-aa@yandex.ru (A.A.K.); 6 Laboratory of Biologically Active Nanostructures, Gamaleya Research Institute of Epidemiology and Microbiology, Gamalei St. 18, Moscow 123098, Russia; gordeev.kirill.loremipsum@gmail.com; 7 Laboratory for Biological Testing and the Mechanism of Action of Biologically Active Substances, G.B. Elyakov Pacific Institute of Bioorganic Chemistry, 159 Prospect 100-Letiya Vladivostoka, Vladivostok 690022, Russia; dminae@mail.ru; 8 Department of Biotechnology and Industrial Pharmacy, Lomonosov Institute of Fine Chemical Technologies, MIREA-Russian Technological University, 86 Vernadsky Prospekt, Moscow 119571, Russia; 4motya@gmail.com; 9 Institute of Medicine, Peoples’ Friendship University of Russia, Miklukho-Maklaya St. 6, Moscow 117198, Russia; 10 Oncology Department, I.P. Pavlov Ryazan State Medical University, Ministry of Health of Russia, Ryazan 390026, Russia

**Keywords:** glucocorticoid, selective glucocorticoid receptor agonist/modulator, lymphoma, leukemia, transrepression, transactivation, osteoporosis, skin atrophy, primary leukemic blasts, synephrine derivatives

## Abstract

Glucocorticoids (GCs) represent effective anti-cancer drugs for the treatment of hematological malignancies, but their clinical use is limited due to their multiple adverse effects. Selective glucocorticoid receptor agonists/modulators (SEGRAMs) modify glucocorticoid receptor (GR) function, shifting it towards therapeutically important transrepression and, therefore, could be safer alternative to GCs. Here we report on the biological activity of four novel glucocorticoid receptor (GR) ligands, derivatives of synephrine, a natural-origin molecule. We demonstrated the affinity of synephrine derivatives in silico and in vitro by molecular dynamics simulation and radioligand binding assay, correspondingly. Further, we tested the induction of apoptosis in cultured cells and cytotoxic effects in primary lymphoblasts from patients with acute lymphoblastic leukemia. Therapeutically important GR transrepression was evaluated by luciferase reporter assay and Q-PCR of transrepression marker genes, while GR transactivation associated with side effects was evaluated by Q-PCR analysis and by the level of GR phosphorylation at Ser211. Anti-cancer effects of the leader compound, 1-[4-(benzyloxy)phenyl]-2-(hexylamino)ethanol (10S-E2), were studied using a murine transplantable lymphoma P388 model. The potential of 10S-E2 to prevent the development of atrophic complication was evaluated using a murine model of glucocorticoid-induced osteoporosis. All studied synephrine derivatives demonstrated high GR affinity, with the IC_50_ value of the most active derivative 10S-E2 being 0.56 µM; the effects on GR function were cell-type-specific. The leader compound, 10S-E2, revealed SEGRAM properties in vitro and demonstrated anti-cancer effects in vivo, inhibiting tumor growth by more than 60%. Although the anti-cancer effect of 10S-E2 was less pronounced than that of the reference drug dexamethasone, non-atrophogenic properties of 10S-E2 make this molecule an attractive candidate for long-term GR-associated therapies.

## 1. Introduction

Glucocorticoids (GCs) are an important class of immunosuppressive drugs used to treat various hematological malignancies and inflammatory diseases [1,2,3]. However, because GC are involved in gluconeogenesis signaling, cell proliferation and apoptosis, and bone formation, the long-term treatment of patients with high-dose GCs is associated with various side effects, including hyperglycemia, type 2 diabetes mellitus, bone and muscle atrophy, osteoporosis, and many other atrophic and metabolic complications [4,5,6,7].

Upon entering the cell, GCs bind to the glucocorticoid receptor (GR), a member of the nuclear receptor superfamily and well-known transcription factor. Ligand binding induces conformational changes and dissociation of the GR multiprotein complex with chaperones. Phosphorylation of specific serine and tyrosine residues and nuclear translocation of GR results in the binding of glucocorticoid-responsive elements (GREs) or negative GREs in gene promoters, enhancers, or silencers, thereby stimulating or suppressing gene transcription (transactivation (TA) or transrepression (TR), respectively) [3,8,9]. Additionally, GR reveals non-genomic effects through protein–protein interactions with pro-proliferative and pro-inflammatory transcription factors (NF-kB, AP-1, STAT family proteins, etc.) following the attenuation of TF activity and DNA-independent TR [10,11,12]. Negative regulation by TR underpins the therapeutic effects while GR TA results in metabolic and atrophic complications, as well as GC resistance [10,12,13].

The development of selective glucocorticoid receptor agonists/modulators (SEGRAMs) was designed to reduce the side effects of GC treatment by separating GR TA and TR activities. Numerous steroidal and non-steroidal SEGRAMs have been described in the literature, including molecules of natural and synthetic origin, with potential applications in the treatment of inflammatory diseases and cancer [5,12,14,15,16,17,18,19,20].

Several SEGRAMs have entered clinical trials, but to date, no drug from the SEGRAM class has reached the market. The development of the leading SEGRAMs, Fosdagrocorat for the rheumatoid arthritis therapy and Mapracorat for the treatment of ocular inflammatory diseases, was put on hold by the companies [21,22,23,24,25,26,27,28,29,30]. Further research is needed in the development of new molecules and expansion of the SEGRAM list, as well as in the clinical evaluation of beneficial and harmful effects.

**Figure 1:**
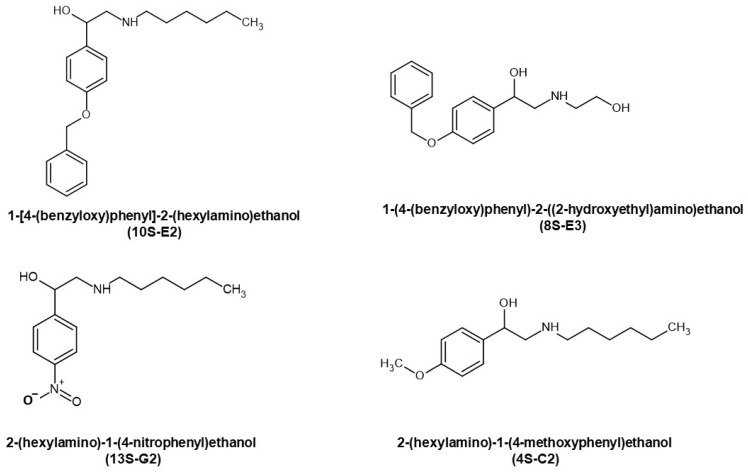
Structures of the synephrine derivatives 4S-C2, 8S-E3, 10S-E2, and 13S-G2.

Recently, we designed and synthesized synephrine derivatives as potential SEGRAMs. Synephrine derivatives could reveal potential clinical benefits in comparison with the effects of GR ligand, selective glucocorticoid receptors agonist CpdA, which is structurally similar to synephrine [31]. Preliminary screening of cytotoxic activity in leukemia and lymphoma cells demonstrated the highest affinity in silico for the most cytotoxic compound, 1-[4-(benzyloxy)phenyl]-2-(hexylamino)ethanol (10S-E2), as well as less toxic synephrine derivatives with the potential to bind GR: 2-(hexylamino)-1-(4-methoxyphenyl)ethanol (4S-C2), 1-(4-(benzyloxy)phenyl)-2-((2-hydroxyethyl)amino)ethanol (8S-E3) and 2-(hexylamino)-1-(4-nitrophenyl)ethanol (13S-G2) (Figure 1 and [32]). The main aim of the present study was to evaluate and discuss the mechanism of the anti-cancer effect of four novel derivatives of synephrine, as well as to study their potential to induce GR-related adverse effects.

Here, we report the molecular mechanisms underlying the in vitro effects of synephrine derivatives and the in vivo moderate anti-cancer activity of the most active compound, 10S-E2. Moreover, we assessed the atrophogenic effect of 10S-E2 in comparison with the glucocorticoid dexamethasone (Dex) in osteoporosis and skin atrophy murine models and demonstrated the absence of atrophogenic potential of the novel synephrine derivative, as opposed to dexamethasone.

## 2. Results

### 2.1. Evaluation of Affinity of Synephrine Derivatives to GR

The affinities of 4S-C2, 8S-E3, 10S-E2, and 13S-G2 for GR were proposed by molecular docking [32]. Here, we aimed to analyze the short-term stability of the complexes of tested compounds with GR by molecular dynamics simulation in silico, as well as to evaluate the binding of 4S-C2, 8S-E3, 10S-E2, and 13S-G2 to GR using a radioligand binding assay in vitro.

**Figure 2:**
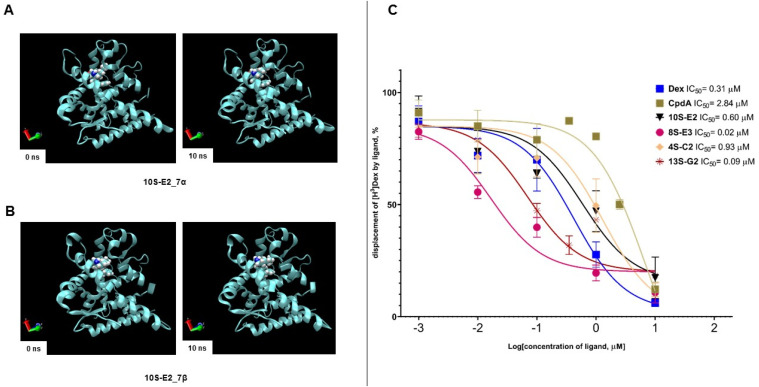
Evaluation of affinity of 4S-C2, 8S-E3, 10S-E2, and 13S-G2 to GR. (**A,B**). Molecular dynamics simulation of the GR complex with 10S-E2_7α (**A**) and 10S-E2_7β (**B**) at the beginning of the trajectory and 10 ns later. The stability of the complex was simulated using molecular dynamics in the GROMACS program. (**C**) GR binding affinity was estimated using a radioligand binding assay. Dex, 4S-C2, 8S-E3, 10S-E2, and 13S-G2 at various concentrations were added to each reaction tube and incubated for 90 min. Cell lysate radioactivity was measured using a RackBeta 1215 LC (LKB Wallac, Turku, Finland) spectrometer for 4 min. The results are presented as the percentage of the individual signal from [3H]Dex. The experiments were performed in triplicate (n = 3).

For the molecular dynamics study, the structures of the stereoisomers of 4S-C2, 8S-E3, 10S-E2, and 13S-G2 at the C-7 position were obtained and minimized (Supplementary Figure S1). Blind molecular docking to GR (PDB ID 1P93) was performed to identify the most favorable complexes of compounds with LBD-GR (Supplementary Table S2, Supplementary Figure S2). The 4S-C2 stereoisomers were predicted not to interact with key Arg611, Gln642, and Thr739 residues in LBD-GR, whereas 8S-E3, 10S-E2, and 13S-G2 may interact with Arg611, Gln642, or Thr739 (Supplementary Table S2, Supplementary Figure S2). Molecular dynamics simulations for 4 ns demonstrated that all ligand–GR complexes were stable (Table 1). The change in the Root Mean Square Deviation (RMSD) of the protein conformation was estimated to be no more than 1.140 Å. Stereoisomers 4S-C2_7α and 4S-C2_7β exhibited the most unstable interactions with GR. For stereoisomers 8S-E3, 10S-E2, and 13S-G2, periodic changes were also observed in the ligand conformations, including those associated with the loss or formation of hydrogen bonds in the complex (Table 1, Supplementary Figures S3–S10). The most promising compound, 10S-E2 [32], was also tested in a 10 ns simulation and was demonstrated to form a stable complex throughout the 10 ns trajectory (Table 1, Figure 2A,B, Supplementary Figures S11 and S12). Two-dimensional molecular docking diagrams demonstrating the interactions between specific functional groups and amino acid residues are shown in Supplementary Figures S13–S20.

In vitro, the synephrine derivative compounds were tested in the concentration range of 0.001–10 µM. We demonstrated that the binding IC_50_ values for the reference GR ligands Dex and CpdA were 0.31 µM and 2.84 µM, respectively, which is in accordance with the literature data, including our previous study [14,33,34,35]. IC_50_ values of 4S-C2, 8S-E3, 10S-E2, and 13S-G2 varied from the micromolar to nanomolar range: 4S-C2–3.21 µM, 8S-E3–0.08 µM, 10S-E2–0.56 µM, and 13S-G2–0.69 µM (Figure 2C). The obtained data indicated that 8S-E3 had the highest affinity in the studied group, and the affinities of 10S-E2 and 13S-G2 were comparable to that of Dex. It should be considered that 10S-E2 and 13S-G2 were the most cytotoxic synephrine derivatives, with cell viability IC_50_ values in the range of 13–26 µM in leukemia and lymphoma cells, while the IC_50_ of 8S-E3 cytotoxic action was in the range of 80–120 µM [32]. At the same time, normal lymphocytes from healthy volunteers were significantly more resistant to the cytotoxic effects of combinational treatments (Supplementary Figure S21). Therefore, 10S-E2, 13S-G2, and 8S-E3 are the most promising GR ligands with anti-cancer effects.

### 2.2. Effects of Synephrine Derivatives on GR Functions

**Figure 3:**
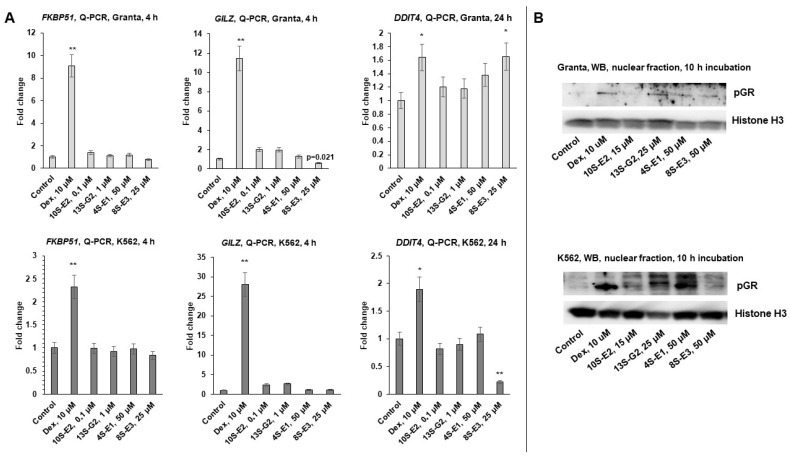
Effects of synephrine derivatives on GR transactivation in Granta lymphoma and K562 leukemia cells. (**A**) Q-PCR analysis of *FKBP51*, *GILZ*, and *DDIT4* mRNA expression in K562 and Granta cells. Cells were treated with Dex (10 µM), 4S-C2 (50 µM), 8S-E3 (25 µM), 10S-E2 (0.1 µM), 13S-G2, or vehicle for 4 h (*FKBP51*, *GILZ*) or 24 h (*DDIT4*). The Q-PCR results were normalized to the expression of the housekeeping gene *RPL27* and are presented as fold changes compared to the control. The mean ± SD was calculated for three individual samples/condition (n = 3). Statistically significant differences compared to the control: * *p* < 0.05, ** *p* < 0.01. (**B**) The levels of the nuclear fraction of phospho-GR (Ser211) were determined using Western blot analysis (n = 3; characteristic images are presented in the figure). Histone H3 was used as a loading control.

To assess the effect of synephrine derivatives 4S-C2, 8S-E3, 10S-E2, and 13S-G2 on GR TA function, we performed Q-PCR analysis of the expression of known GR-target genes, including *FKBP51*, *GILZ*, and *DDIT4* [19,36,37,38,39,40], after incubation for 4 h for *FKBP51* and *GILZ* genes and 24 h for the *DDIT4* gene with the compounds under study or Dex as a reference drug. Depending on the treatment duration and cell line, Dex induced a 5-30-fold increase in the expression of TA marker genes, whereas compounds 10S-E2 and 13S-G2 did not increase the expression of the abovementioned genes (Figure 3A). The effects of 4S-C2 and 8S-E3 were cell-specific: 8S-E3 strongly attenuated *DDIT4* expression by more than three times in K562 cells but induced a 1,5-fold decrease in this gene expression in Granta cells. These results could be associated with cell-dependent GR cofactors and require further investigation and adjustment of the experimental framework (Figure 3A). Q-PCR results were confirmed by Western blot analysis of the level of GR phosphorylated by Ser211, known as a crucial protein modification for GR TA realization because of its binding to short cysteine-rich fragments of the coactivator CREB-binding protein [41]. Treatment of the cells with Dex as a comparator and compounds 4S-C2, 8S-E3, and 13S-G2 induced a weak increase in the GR phosphorylation level, whereas 10S-E2 did not affect the phosphorylation of GR by Ser211, which was in accordance with Q-PCR data (Figure 3B, Supplementary Figure S22).

**Figure 4:**
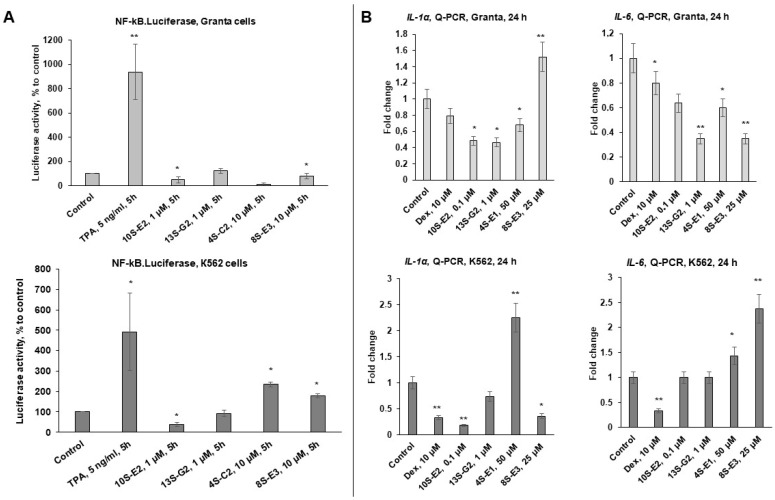
Effects of synephrine derivatives on GR transrepression in Granta lymphoma and K562 leukemia cells. (**A**) K562 and Granta cells stably infected with the lentiviruses bearing the NF-kB luciferase reporter. Cells were incubated for 5 h with the solvent (control), TPA (5 ng/mL), 4S-C2 (10 µM), 8S-E3 (10 µM), 10S-E2 (1 µM), or 13S-G2 (1 µM). Luciferase activity was determined as described in the Materials and Methods section. (**B**) *IL1-α* and *IL6* mRNA expression in K562 and Granta cells. Q-PCR results were normalized to the expression of the housekeeping gene *RPL27* and are presented as the fold change compared to the control. The mean ± SD was calculated for three individual samples/condition (n = 3). Statistically significant differences compared to the control: * *p* < 0.05, ** *p* < 0.01.

We analyzed GR TR function using an NF-kB.Luc reporter assay with the irritating inflammatory agent phorbol 13-acetate 12-myristate (TPA) as an inducer of NF-kB signaling and positive control. We found that TPA induced 5-9-fold NF-kB activation, while 10S-E2 was the most effective in reducing NF-kB activity in K562 cells, inhibiting luciferase activity by 60%. In Granta cells, 10S-E2 inhibited luciferase activity by 15%, but this decrease was not statistically significant. Data on 4S-C2 and 8S-E3 were cell-specific; thus, 4S-C2 significantly decreased NF-kB activity in Granta cells but induced it in K562 cells (Figure 4A).

The 10S-E2 effects on NF-kB signaling were further confirmed by Q-PCR analysis, demonstrating the downregulation of the following GR TR marker genes: interleukins *IL1-α* and *IL6* involved in lymphoid cell survival and inflammatory response [36,42,43,44] (Figure 4B).

### 2.3. Pro-Apoptotic and Anti-Cancer Effects of Synephrine Derivatives In Vitro and In Vivo

**Figure 5:**
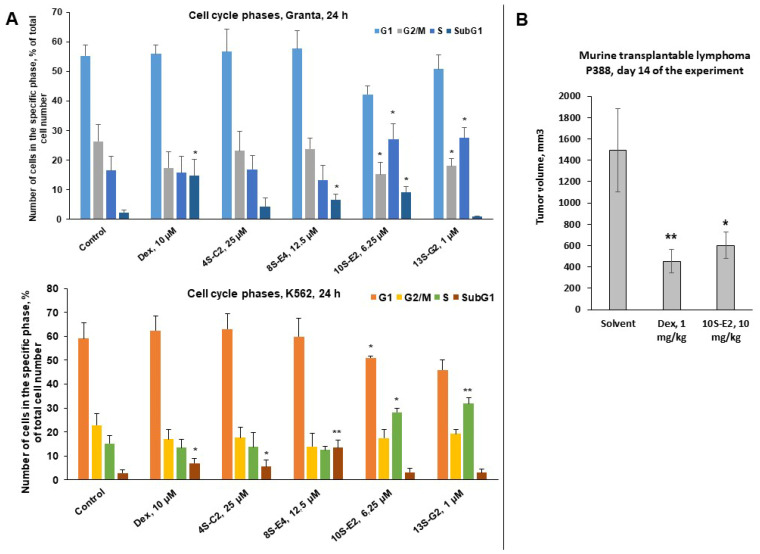
Pro-apoptotic and anti-cancer effects of synephrine derivatives in vitro and in vivo. (**A**) Effect of 4S-C2, 8S-E3, 10S-E2, and 13S-G2 on cell cycle progression in K562 and Granta cells after 24 h of incubation. The cells were fixed with ethanol, stained with propidium iodide, and analyzed using flow cytometry. Statistically significant difference compared to control: *—*p* < 0.05, **—*p* < 0.01 (n = 3). (**B**) In vivo anti-cancer activity was evaluated using the transplantable P388 murine lymphoma model. Animals were treated i.p. 3 times per week with Dex (1 mg/kg), 10S-E2 (10 mg/kg), or solvent (ethanol/tween 80/distilled water = 0.5:0.5:9). Tumor nodule size was measured twice a week using digital calipers. Statistically significant differences compared to the control: * *p* < 0.05, ** *p* < 0.01.

We examined the effects of 4S-C2, 8S-E3, 10S-E2, and 13S-G2 on cell cycle progression and apoptosis induction using flow cytometry. Cell cycle analysis showed that the level of apoptosis (the sub-G1-phase cell population) in Granta cells treated with the synephrine derivatives and Dex was higher than that in the control samples (Figure 5A). In the case of 10S-E2 treatment, a decrease in the number of cells in the G1 phase was observed in both cell lines. Interestingly, 10S-E2 did not induce apoptosis in K562 cells but increased the number of replicating cells in the S phase. This could reflect the different origins of the cells, followed by fluctuations in signaling and response to treatment.

Overall, 10S-E2 was the most active compound among the studied synephrine derivatives and was tested in pilot experiments in vivo on a murine transplantable tumor lymphoma P388 model (Figure 5B) and ex vivo using primary leukemic blasts from pediatric patients with acute lymphoblastic leukemia (ALL) (Table 2). We found that the inhibition of tumor growth in the animals treated with Dex (1 mg/kg) and 10S-E2 (10 mg/kg) was approximately 60–70%, with Dex showing superiority (Figure 5B). Toxicity was monitored through general cage observations and weight monitoring. Dex was used in a well-established therapeutic and non-toxic dose in mice ([14,45,46] and Supplementary Figure S23). The IC_50_ values of 10S-E2 evaluated in primary lymphoblast samples from 19 patients diagnosed with ALL ranged from 40 to 70 µM (Table 2). This could reflect the lower sensitivity of lymphoblasts derived from patients. Dosage regimens should also be developed and optimized.

### 2.4. 10S-E2 Did Not Induce Atrophic Changes in Skin and Bone Tissue Compared to Dex

**Figure 6:**
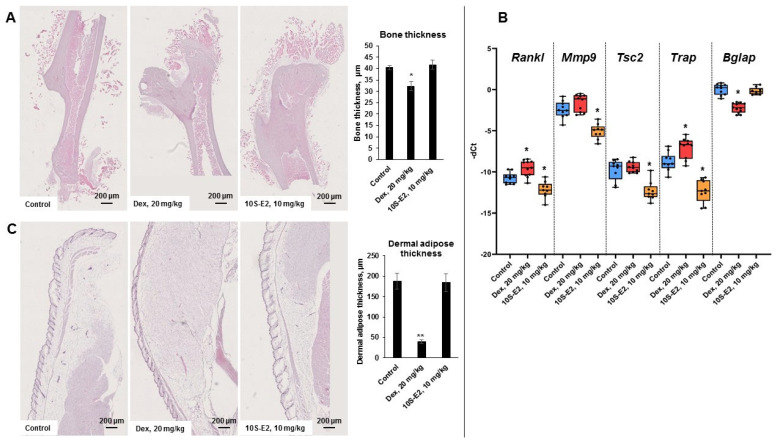
10S-E2 did not induce atrophic changes in skin and bone tissue compared to Dex; 10 week-old female BALB/c mice (10 animals/group) were treated every 24 h with i.p. injections for 5 weeks with Dex (20 mg/kg), 10S-E2 (10 mg/kg), and the solvent (ethanol/tween 80/distilled water = 0.5:0.5:9). (**A**) H&E bone staining and quantification of cortical bone thickness. (**B**) Q-PCR analysis of *Rankl*, *Mmp9*, *Tsc2*, *Trap*, and *Bglap* mRNA expression in bone tissue. The Q-PCR results (for three individual RNA samples/condition, n = 3) were normalized to the expression of the housekeeping gene *Rpl27* and presented as fold change compared to the control. Control (blue), Dex (red), 10S-E2 (orange). (**C**) H&E skin staining and quantification of dermal adipose thickness (50 images per treatment group of 10 animals). Statistically significant differences compared to the control: * *p* < 0.05, ** *p* < 0.01.

We selected 10S-E2, which most significantly modulated GR function and displayed anti-cancer effects both in vitro and in vivo, to test its possible atrophogenic effect in skin and bone. 10S-E2 is a GR ligand, and the activated receptor could act in a pleiotropic manner depending on the tissue affected [37,45,47,48]. To test whether 10S-E2 could avoid the atrophogenic action of GC, we optimized a previously described model of GC-induced osteoporosis in Balb/c female mice [45,49,50], formed the group of animals with pathology, and assessed atrophy in both bone and skin induced by high-dose i.p. Dex injections (20 mg/kg), which were administered every 24 h for 5 weeks. 10S-E2 (10 mg/kg) or vehicle was injected into animals in the experimental and control groups. Animal weight was similar between the control and experimental groups (Supplementary Figure S24). We observed a significant decrease in cortical bone thickness between animals in the control and Dex-treated groups, whereas 10S-E2 did not cause any loss in cortical bone thickness (Figure 6A) compared to the Dex-induced bone thickness loss of 30%. Moreover, this observation was supported with Q-PCR data on the expression of known osteoporosis and cellular matrix degradation marker genes: *Rankl* (associated with bone resorption) [51], *Trap* (marker of osteoclast activity), *Mmp9* (cellular matrix degrader) [52,53], *Tsc2* (hypoxia-induced negative regulator of proliferation) [54], and *Bglap* (osteogenic marker) [55,56]. Downregulation of *Bglap*, upregulation of *Rankl* and *Trap*, and a tendency toward *Mmp9* and *Tsc2* upregulation clearly proved osteoporosis development in Dex-treated animals (Figure 6B). In contrast, 10S-E2 significantly downregulated *Rankl*, *Tsc2*, *Mmp9*, and *Trap* compared to Dex and the control (Figure 6B).

To test whether 10S-E2 could induce the development of skin atrophy as a GR-activating ligand, we assessed morphological changes in the skin of Balb/c female mice. In agreement with previously published results [45], prolonged treatment with systemic Dex led to a significant cutaneous atrophy, with dermal adipose tissue reduced by approximately 70% (Figure 6C). At the same time, 10S-E2 preserved the skin architecture, and it remained similar to that of the control group (Figure 6C).

## 3. Discussion

Glucocorticoids are involved in the regulation of different physiological processes modulating numerous and multidirectional signals. A search for GC alternatives with a similar spectrum of clinically demanded anti-proliferative and anti-inflammatory actions but with attenuated side effects is urgently needed for thousands of patients. The preservation of therapeutically important GR TR could be achieved by using a combination of GCs with tissue protectors against metabolic and atrophic complications [16,36,37,40]. Another option assumes the design of selective glucocorticoid receptor agonists/modulators (SEGRAMs) that shift GR activity toward TR and could serve as GC alternatives with an improved therapeutic index, referring to the ratio between the mean toxic dose associated with side effects and the effective dose [16,18,57,58,59,60,61,62,63,64].

Previously, we developed and described derivatives of Compound A (CpdA), a widely characterized molecule from the SEGRAM class, 4-(1-hydroxy-2-(piperidin-1-yl)ethyl)phenol or CpdA-03, which demonstrated superior biological activity compared to CpdA [14]. The second strategy for the development of novel SEGRAMs was the use of the synephrine molecule as a template for SEGRAM design [32]. An approach to the development of novel SEGRAMs using the templates of compounds of natural origin, with anti-inflammatory activity comparable to that of GC, was described for boswellic acids and their derivatives. These boswellic acid derivatives originated from natural sources without any synthetic modification of the boswellic acid molecule [65]. Our approach, based on the chemical modification of naturally occurring proven or potential SEGRAMs, is still pioneering in the field of novel partial GR agonists.

Preliminary screening of the cytotoxic effects revealed 10S-E2 and 13S-G2 as leader compounds with the highest affinity to GR in silico and in vitro (Figure 2 and [32]), which was in line with the pronounced inhibitory effects on the expression of GR-regulated proliferative and inflammatory genes (GR TR, Figure 4) and the absence of GR TA induction (Figure 3). The divergent effects of 8S-E3, 4S-C2, and 13S-G2 on gene expression patterns, as well as their moderate cytotoxic activity, make these three compounds less promising for further analysis. Therefore, 10S-E2 is the most prominent molecule for further preclinical studies and pharmaceutical development. It has to be mentioned that the difference between the GR binding affinity of 10S-E2 and its cytotoxic potency is significant. As both cell lines are well characterized by high GR expression [19], the difference in affinity and cytotoxic action could be associated with the permeability and distribution of synephrine derivatives in cells. Activators of cell permeability could be an option.

Furthermore, it was demonstrated that the lead compound, 10S-E2, exhibited cytotoxic effects at concentrations of 10–20 μM in mantle cell lymphoma Granta and chronic myeloleukemia K562 cells and at concentrations of 40–70 μM in primary PBMC from patients with ALL, together with the induction of apoptosis in Granta cells (Figure 5 and [32]), which exceeded the moderate cytotoxicity of the template molecule, synephrine [66,67,68]. The anti-cancer effects of 10S-E2 in vivo, compared to the reference drug Dex, could be associated with GR TR activation and did not lead to the development of GC-induced resistance [69,70]. Moreover, the absence of atrophic effects in the glucocorticoid-induced osteoporosis model demonstrated the safety profile of the molecule (Figure 6). Therefore, the present study reveals the advantage of 10S-E2 as a GR ligand with anti-cancer properties and a higher safety profile. Similar studies of protective effects were earlier reported for ginsenoside Rg1, a selective glucocorticoid receptor agonist of natural origin [28,29]. Further studies should be conducted to evaluate the possible protective effects of 10S-E2 individually and in combination with GCs, to expand the panel of models with other leukemia and lymphoma cells including cells with GR knockdown, as well as with models in vivo. Moreover, all tested compounds present the mixture of enantiomers used for the pilot evaluation of biological activity. The synthesis of enantiopure 10S-E2 isomers and the comparative analysis of their biological effects will be an important task to take on. As GC-related side effects are complex and affect multiple physiological processes, the panel of GC-dependent adverse effect models should be expanded, in particular, in the study of metabolic complications. A comparative analysis of GCs and 10S-E2 activities in models of metabolic side effects should be performed.

## 4. Materials and Methods

### 4.1. Cells and Treatments

The chronic myeloid leukemia cell line K562 and mantle cell lymphoma cell line Granta were obtained from ATCC and cultured in RPMI-1640 medium (“Paneco”, Moscow, Russia) supplemented with a 10% fetal bovine serum (“Biowest”, Nuaille, France), 2 mM L-glutamine, 5 ME/mL penicillin, and 5 µg/mL streptomycin (“Paneco”, Moscow, Russia) at 37 °C and 5% CO_2_ as described [19].

Cells were treated with dexamethasone (Dex) (“Macklin”, Shanghai, China), CpdA, 4S-C2, 8S-E3, 10S-E2, and 13S-G2 synthesized as described [32], or vehicle (0.01% DMSO unless other specified).

### 4.2. Cell Cycle Analysis

The distribution of cells by cell cycle phases was evaluated using propidium iodide (PI) staining, as described in [19]. Cells were seeded into 24-well plates (10^5^ cells/well) and treated with Dex (50 µM), 4S-C2 (25 µM), 8S-E3 (12.5 µM), 10S-E2 (6.25 µM), 13S-G2 (1 µM), or the vehicle for 24 h. After incubation, the cells were resuspended in 70% ethanol and fixed for 2h at 4 °C. Cells were then washed twice with cold PBS, pH 7.4, and stained with 500 µL of cold propidium iodide (PI) solution (50 µg/mL PI, 1% Triton X-100, and 100 µg/mL RNase A in PBS). The cell cycle distribution of cells in samples was analyzed using an FACSCalibur Flow Cytometer (“BD Biosciences”, San Jose, CA, USA). The experiments were performed in triplicate.

### 4.3. Western Blot Analysis

Cells were seeded into 24-well plates (10^5^ cells/well) and treated with Dex (10 µM), 4S-C2 (50 µM), 8S-E3 (50 µM), 10S-E2 (15 µM), 13S-G2 (25 µM), or the vehicle for 10 h. Western blot analysis was performed as previously described [19,71]. Proteins were resolved using SDS-PAGE. Membranes were blocked with 5% nonfat milk solution and then incubated with primary antibodies against phosphorylated GR (pGR, Ser211) (“Santa Cruz Biotechnology”, Santa Cruz, CA, USA) overnight at 4 °C. To verify equal protein loading and adequate transfer, the membranes were probed with Histone H3 antibodies (“Cell Signaling”, Boston, MA, USA). Goat anti-rabbit IgGs (“Jackson Immuno Research”, West Grove, PA, USA) were used as secondary antibodies. Signals were detected using ECL reagent and an ImageQuant LAS4000 system (“GE HealthCare”, Chicago, IL, USA). ImageJ 1.54h software was used for densitometry. The experiments were performed in triplicate.

### 4.4. Human Peripheral Blood Mononuclear Cell (PBMC) Isolation and Culture

Peripheral blood samples were collected from 1–10-year-old pediatric patients with newly diagnosed ALL in the acute phase of the disease or from adult healthy volunteers at N.N. Blokhin National Research Center of Oncology. The Ethical Committee of the N.N. Blokhin National Research Center of Oncology approved this study, and written informed consents were provided. Monocytes were isolated by centrifugation with Ficoll-Isopaque (“Paneco”, Russia) and then cultured in the RPMI-1640 media (“Paneco”, Russia) supplemented with 20% FBS (“Biowest”, France), 2 mM L-glutamine, 0.5 ME/mL penicillin and 0.5 µg/mL streptomycin (“Paneco”, Russia) and 10 mg/L phytohaemagglutinin (“Paneco”, Moscow, Russia).

### 4.5. Resazurin Cytotoxicity Assay

Isolated PBMCs were seeded into 96-well plates (2 × 10^6^ cells/well) and treated with 10S-E2 at a wide range of concentrations (2–200 µM). Cell viability was analyzed after 24 h of incubation, assessing metabolic activity with the addition of 0.177 mg/mL of resazurin at 8–10 h before the end of the incubation. Fluorescence intensity was evaluated using a Fluoroskan FL microplate fluorimeter (“Thermo Scientific”, Waltham, MA, USA). The IC_50_ values were determined using GraphPad Prism (Ver.7.0) software. The experiments were performed in triplicate.

### 4.6. RNA Extraction and Q-PCR

Total RNA isolation from cell cultures and murine bones, reverse transcription, and Q-PCR were performed as described in [36]. Primers were designed using NCBI Primer-BLAST https://www.ncbi.nlm.nih.gov/tools/primer-blast/, accessed on 25 August 2025 (Supplementary Table S1). Results were normalized to the expression of the housekeeping *RPL27* и *Rpl27* genes [36,37]. The experiments were performed in triplicate.

### 4.7. Lentivirus Preparation and Cell Transduction

Lentiviral stocks were generated using the lentiviral expression vector NF-kB. Luc was encoded with Firefly Luciferase reporters with the NF-kB-responsive promoter of minimal CMV promoter as a control (“System Bioscience”, Moutain View, CA, USA), along with packing plasmids GAG, REV, and VSVg [14,72]. HEK-293T cells were transfected with the abovementioned plasmids using TurboFect reagent (“Thermo Scientific”, Waltham, MA, USA) according to the manufacturer’s protocol. Viral stocks were collected after 24–48 h after transfection, sterilized by 0.2 µm filtration, and applied to K562 and Granta cells for 24 h. K562 and Granta cells infected with lentiviruses were selected using 0.5 µg/mL puromycin. The infection efficiency exceeded 90% (tracked by GFP fluorescence).

### 4.8. Luciferase Assay

Cells (10^4^ cells/well in a 24-well plate), stably expressing Firefly Luciferase under the NF-kB promoter, were treated for 5 h with TPA (12-O-tetradecanoylphorbol-13-acetate, Boston, MA, USA, 10 ng/mL), 4S-C2 (10 µM), 8S-E3 (10 µM), 10S-E2 (1 µM), 13S-G2 (1 µM), or the vehicle. Luciferase activity was measured as described [36,37] using a commercial Luciferase Assay (“Promega”, Madison, WI, USA) and Luminometer TD 20/20 (“Turner Design Instruments”, San Jose, CA, USA). Cells stably expressing Firefly Luciferase under the minimal CMV promoter were used as a control to adjust for nonspecific toxicity. The results were normalized to the total protein amount in each sample. The experiments were performed in triplicate.

### 4.9. Molecular Docking and Molecular Dynamics Simulation

Ligand structures were prepared using ChemDraw 17.0 software. Molecular docking was performed using the SwissDock online server (http://old.swissdock.ch, assessed on 25 August 2025), and the results were analyzed using UCSF Chimera 1.16. Blind docking resulted in 250 models for each compound, which were grouped into 35–45 clusters. Cluster 0 was selected for further analysis. The stability of the complex was studied using molecular dynamics simulations in GROMACS 2023.3. The loading file for the calculations was prepared using the CHARMM-GUI generator (https://www.charmm-gui.org). A constant pressure of 1 atm, a temperature of 313 K, and a CHARMM force field were used. Because the binding of the receptor to the ligand occurs in the cytoplasm, basic solution conditions were selected for the simulation. Molecular dynamics calculations in GROMACS were performed with a time step of 0.002 ps, and the coordinates were recorded every 5000 steps. The trajectory lengths were 4 and 10 ns. The obtained trajectories were analyzed using VMD 2.0.0a7. Two-dimensional diagrams of ligand–protein interactions were obtained using BIOVIA Discovery Studio Visualiser v.25.1.0.24284.

### 4.10. GR Binding Affinity Assay

K562 cells (2.5 × 10^6^ cells/mL) were cultured in a serum-free culture medium in the presence of 0.5 µM [3H]Dex and unlabeled Dex, CpdA, 4S-C2, 8S-E3, 10S-E2, and 13S-G2 at various concentrations. After 90 min of incubation, the cells were washed twice by centrifugation in cold serum-free medium (1500 rpm, 5 min). The cells were then lysed with RIPA buffer (50 mM Tris (pH 8.0), 150 mM NaCl, 1 mM ethylenediaminetetraacetic acid, 0.1% sodium dodecyl sulfate, 0.5% sodium deoxycholate, 1% triton-X100, 10% glycerin, and protease inhibitor) for 20 min. Lysates were transferred to scintillation liquid, and radioactivity was measured using a RackBeta 1215 LC spectrometer for 4 min. The relative binding percentages of Dex, CpdA, 4S-C2, 8S-E3, 10S-E2 and 13S-G2 were measured. IC_50_ was determined using GraphPad Prism software, Ver.7.0. Measured values represented in decay per minute (dpm) of different concentrations of Dex were calculated using a method of non-linear regression (curve fit) with the following equation: [Inhibitor] vs. response (three parameters: “top”, “bottom”, and IC_50_). “Top” meant the highest value in dpm and was equal to 100%, while the “bottom” was considered as the lowest value (0%). Dpm values for 4S-C2, 8S-E3, 10S-E2, and 13S-G2 were calculated in the same way using the “bottom” of Dex in the individual biological repeat. These calculations led to the plotting curves that most precisely fit the pattern of competitive inhibition by a ligand of binding [3H]Dex to GR. The IC_50_ values for each ligand were determined by plotting these curves. The experiments were performed in triplicate.

### 4.11. Anti-Cancer Study In Vivo

The protocol for the experiment was approved by the local N.N. Blokhin National Medical Research Center Ethics Committee and corresponded to the guidelines for the welfare and use of animals in cancer research adopted by The United Kingdom Coordinating Committee on Cancer Prevention Research [73]. P388 cells were injected i.p. into 5-week-old female DBA/2 mice (“Stolbovaya Farm”, Stolbovaya, Russia). After 15 days, the cells were collected from the peritoneum, washed, and resuspended in PBS. For the experiments, 0.1 mL containing 2 million cells obtained from the ascites was inoculated s.c. in 5-week-old female BDF1 mice (“Stolbovaya Farm”, Stolbovaya, Russia). Mice were randomly divided into three groups, with 10 animals per group, and treated i.p. 3 times/week with Dex (1 mg/kg), 10S-E2 (10 mg/kg), or the solvent (ethanol/tween 80/distilled water = 0.5:0.5:9). Body weight was recorded twice a week for toxicity determination. Tumor size was measured three times per week using digital calipers.

### 4.12. Glucocorticoid-Induced Osteoporosis

The protocol for the experiment was approved by the local N.N. Blokhin National Medical Research Center Ethics Committee and corresponded to the guidelines for the welfare and use of animals in cancer research adopted by The United Kingdom Coordinating Committee on Cancer Prevention Research [73]. Specifically, 10-week-old BALB/c female mice (“Stolbovaya-farm”, Stolbovaya, Russia) were randomly divided into three groups, with 10 animals per group, and treated i.p. every 24 h for 5 weeks with Dex (20 mg/kg) for glucocorticoid-induced osteoporosis (GIOP) as described [49], 10S-E2 (10 mg/kg), and the solvent (ethanol/tween 80/distilled water = 0.5:0.5:9). Body weight was recorded twice a week for toxicity control.

### 4.13. Histology and Morphometry

Bone and skin samples from the GIOP experiment were fixed in formaldehyde and embedded in paraffin, and sections were stained with hematoxylin and eosin. After formaldehyde fixation, the bone samples were decalcified in decalcification buffer (0.5 M EDTA, 0.7 mM NaOH, pH7.0) for 14 days, washed twice with PBS, and further processed according to the standard histology protocol. The dermal adipose and bone thickness were quantified in sections stained with hematoxylin and eosin. At least five individual fields per slide of each sample/treatment (at least 50 images/treatment group) were analyzed using Axioplan2 microscope software (“Carl Zeiss”, Jena, Germany). The dermal adipose or bone thickness in treated animals is presented as a percentage of the control animals.

### 4.14. Statistical Analysis

Mean and standard deviation values were calculated using Microsoft Excel and GraphPad Prism (Ver.7.0) software. The treatment effects in each experiment were compared using a one-way ANOVA or *t*-test. Differences between groups were considered significant at *p* < 0.05.

## 5. Conclusions

In this study, we investigated the biological effects of novel GR ligands from the class of synephrine derivatives and demonstrated the anti-cancer effect of 1-[4-(benzyloxy)phenyl]-2-(hexylamino)ethanol (10S-E2) in vitro, in vivo, and ex vivo. Moreover, for 10S-E2, we showed the absence of GR transactivation, resulting in the preservation of bone and skin tissues during long-term treatment. This fact shows the advantage of 10S-E2 as a GR ligand with anti-cancer properties and a higher safety profile, and we propose combining this synephrine derivative with GCs to study the potential protective effect of 10S-E2.

## Figures and Tables

**Table 1:** RMSD data of complexes of synephrine derivatives with GR.

Time of Simulation	Compound								
4 ns		Protein				Ligand			
		avr	sd	min	max	avr	sd	min	max
	4S-C2_7α	1.043	0.048	0.399	1.128	0.822	0.125	0.138	1.194
	4S-C2_7β	1.048	0.049	0.395	1.134	0.883	0.100	0.228	1.139
	8S-E3_7α	1.055	0.055	0.388	1.150	0.474	0.067	0.09	0.678
	8S-E3_7β	1.042	0.044	0.4	1.112	0.488	0.094	0.139	0.860
	10S-E2_7α	1.042	0.042	0.387	1.106	0.565	0.117	0.120	0.999
	10S-E2_7β	1.057	0.073	0.007	1.140	0.497	0.079	0.095	0.769
	13S-G2_7α	1.062	0.049	0.392	1.136	0.550	0.122	0.140	0.886
	13S_G2_7β	1.060	0.042	0.378	1.120	0.561	0.118	0.168	0.932
10 ns		Protein				Ligand			
		avr	sd	min	max	avr	sd	min	max
	10S-E2_7α	1.070	0.041	0.387	1.157	0.319	0.081	0.110	0.707
	10S-E2_7β	1.089	0.041	0.397	1.175	0.550	0.118	0.100	0.939

**Table 2:** The IC_50_ values of 10S-E2 cytotoxic effects in primary lymphoblasts from pediatric patients with acute lymphoblastic leukemia.

No	Patient Code	IC_50_, μM
1	3	40.9 ± 3.12
2	4	43.2 ± 9.48
3	7	43.0 ± 0.71
4	8	60.6 ± 0.97
5	10	71.3 ± 5.59
6	12	75.7 ± 4.63
7	20	64.2 ± 0.55
8	21	49.4 ± 1.95
9	23	55.2 ± 2.25
10	24	70.3 ± 2.54
11	25	80.3 ± 4.09
12	30	52.4 ± 0.07
13	31	63.2 ± 1.42
14	33	67.6 ± 4.02
15	34	55.0 ± 1.91
16	35	65.9 ± 0.27
17	37	69.8 ± 3.66
18	39	71.3 ± 2.59
19	46	71.5 ± 2.08

## Data Availability

The original contributions presented in this study are included in the article/Supplementary Materials. Further inquiries can be directed to the corresponding authors.

## Supplementary Materials

The supporting information can be downloaded at: https://www.mdpi.com/article/10.3390/ijms262311404/s1.

## Abbreviations

The following abbreviations are used in this manuscript:

ALL	acute lymphoblastic leukemia
Bglap	bone gamma-carboxyglutamic acid-containing protein
CMV	cytomegalovirus
CpdA	compound A
DDIT4	DNA-damage-inducible transcript 4
Dex	dexamethasone
FKBP51	FK506-binding protein 51
GC	glucocorticoid
GILZ	glucocorticoid-induced leucine zipper
GIOP	glucocorticoid-induced osteoporosis
GR	glucocorticoid receptor
GRE	glucocorticoid-responsive element
H&E	hematoxylin and eosin
HEK	human embryonic kidney
IC_50_	50% inhibitory concentration
IL1-α	interleukin 1-α
IL6	interleukin 6
Mmp9	matrix metalloproteinase-9
PBMC	peripheral bone marrow cell
Q-PCR	quantitative polymerase chain reaction
Rankl	receptor activator of nuclear factor kappa-Β
RMSD	root mean square deviation
RPL27	ribosomal protein 27
SEGRAM	selective glucocorticoid receptor agonist/modulator
TA	transactivation
TPA	12-O-tetradecanoylphorbol-13-acetate
TR	transrepression
Trap	tartrate-resistant acid phosphatase
TSC2	tuberoses sclerosis complex 2
