# Supplementary material for: Anti-Cancer Outcome of Glucocorticoid Receptor Transrepression by Synephrine Derivatives in Hematological Malignancies
Source: Int J Mol Sci. 2025 Nov 25;26(23):11404. doi: 10.3390/ijms262311404 (PMC12691742; doi:10.3390/ijms262311404)
Supplement: Supplementary file 1 [file ijms-26-11404-s001.zip › ijms-3972422-supplementary/Zhidkova et al Supplementary Material Revised3.pdf]

Supplementary Table S1. Primer sequences for Q-PCR analysis

Gene symbol	Sequence	
	Forward 5’-3'	Reverse 5’-3'
**human**		
RPL27	ACCGCTACCCCCGCAAAGTG	CCCGTCGGGCCTTGCGTTTA
FKBP51	GAATGGTGAGGAAACGCCGAT	TGCCAAGACTAAAGACAAATGGT
GILZ	AACAACGAAATGTATCAGACCC	TGTCCAGCTTAACGGAAACCA
DDIT4		
IL1α	ATGATGGCTTATTACAGTGGCAA	GTCGGAGATTCGTAGCTGGA
IL6	GGGAGCGATAAACACAAACTCTGC	GAGAAGGCAACTGGACCGAAG
**mouse**		
Rpl27	G	T
Rankl		
Tsc2		
Bglap		
Mmp9	CGCTCATGTACCCGCTGTAT	
Trap		

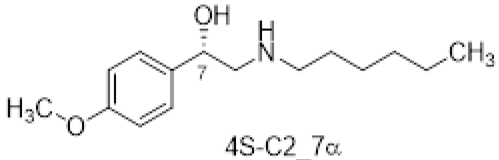

4S-C2_7α

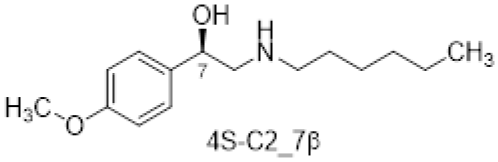

4S-C2_7β

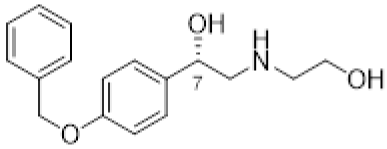

8S-E3_7α

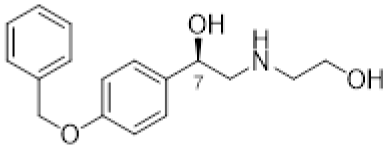

8S-E3_7β

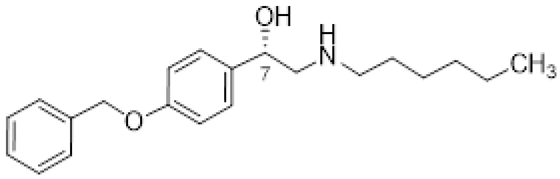

10S-E2_7α

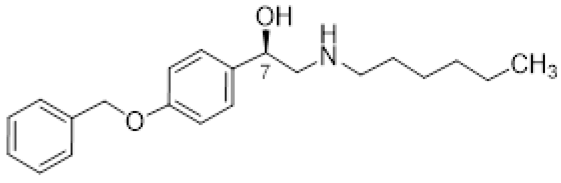

10S-E2_7β

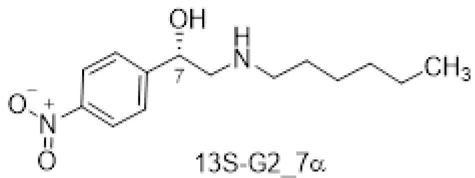

13S-G2_7α

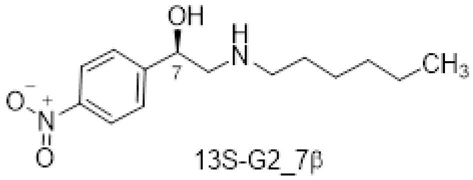

13S-G2_7β

Supplementary Figure S1. Structures of 4S-C2, 8S-E3, 10S-E2, 13S-G2 stereoisomers

Supplementary Table S2. Molecular docking results

Compound	ΔG, kcal/mol	, kcal/mol	Hydrogen binding, Å	Hydrophobic interactions
Dex			Arg611 … O, 2.146<br>H27 … Gln642, 2.351<br>H26 … Thr739, 2.115	Met560, Leu566, Gly567, Trp600, Met601, Met604, Phe623, Met646, Tyr735, Cys736, Thr739, Ile747
S-			H11 Asn564, 2.061<br>H10 Asn564, 2.053	Met560, Met646, Met601, Met604, Leu563, Leu753
S-C2	-7.944454	-19.7369	H10 … Asn564, 2.191<br>H11 … Asn564, 2.483	Gly567, Met604, Phe623, Trp600, Cys736, Leu563, Phe749, Met560, Cys643, Met646
S-E3_α	-8.359835	0.940063	H15 … **Gln642,** 1.893<br>H14 … Leu732, 2.802<br>Asn564 … O2, 2.186	Phe749, Thr739, Tyr735, Leu732, Met560, Met646, Leu608, Phe623, Gly567
8S-E3_	-8.500033	4.746	**Arg611** … O2, 2.371<br>H20 … Gln570, 2.579<br>Leu563 … H15, 2.897	Gly567, Leu563, Phe623, Met646, Met601, Cys736, Tyr735, Leu732, Thr739, Trp600
10S-	-8.699412	-10.2798	H14 … Asn564, 2.036<br>H15 … **Gln642,** 2.106	Met639, Met560, Cys643, Met646, Phe623, Leu563, Met601, Gly567, Leu608, Met604, Phe623, Phe749
10S-	-8.176007	-3.67274	Asn564 … O1, 2.109<br>H14 … Asn564, 2.034	Gly567, Leu566, Leu608, Met604, Phe623, Trp600, Met646, Leu732, Tyr735, Leu563, Met560, **Thr739**
13S-	-8.123218	-26.5631	**Arg611** … O1, 1.902	Leu608, Met604, Leu566, Phe623, Leu563, Met601, Leu732, Tyr735, Cys736, Thr739, **Ile747**, Met560
13S_	-8.295626	-22.0501	**Arg611** … O1, 2.779<br>H8 … Asn564, 1.808	Phe749, Cys736, Met646, Phe623, Leu608, Ala605, Met604, Leu753, Met560

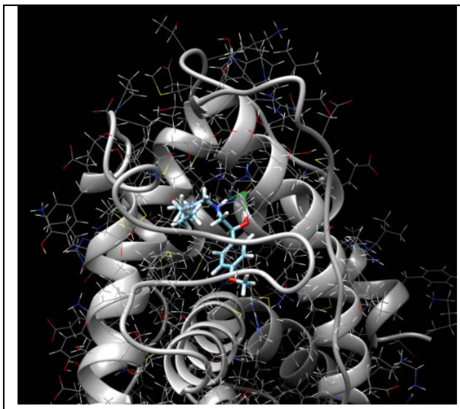	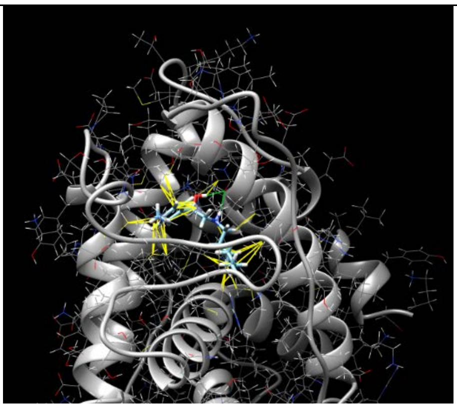
S	S
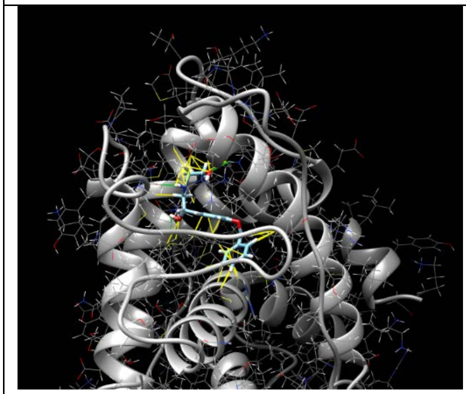	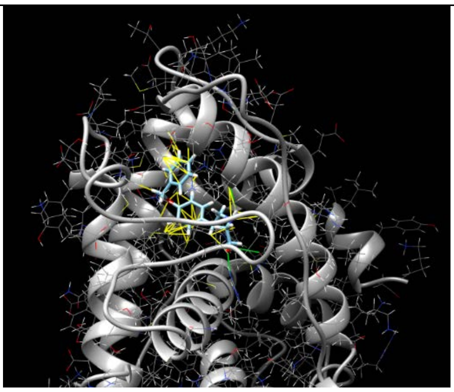
S	8
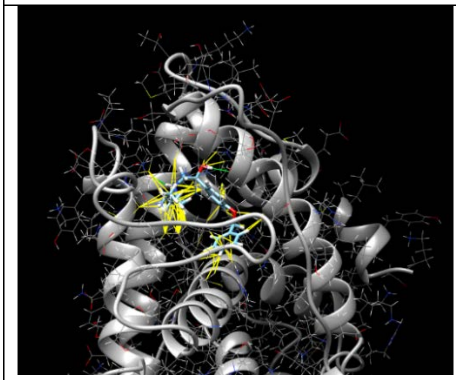	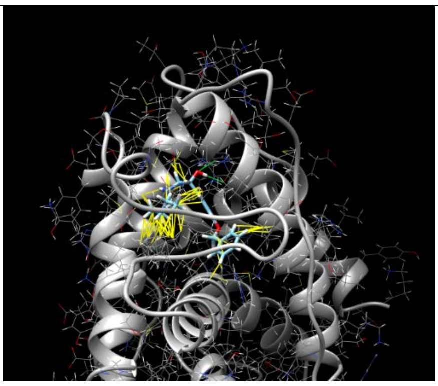
1	1
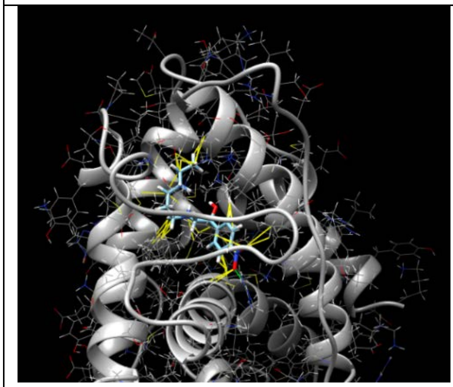	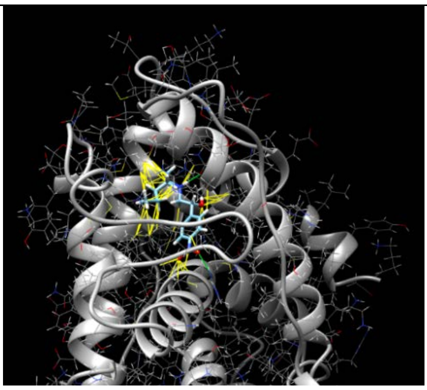
1	1

Supplementary Figure S2. Molecular docking results

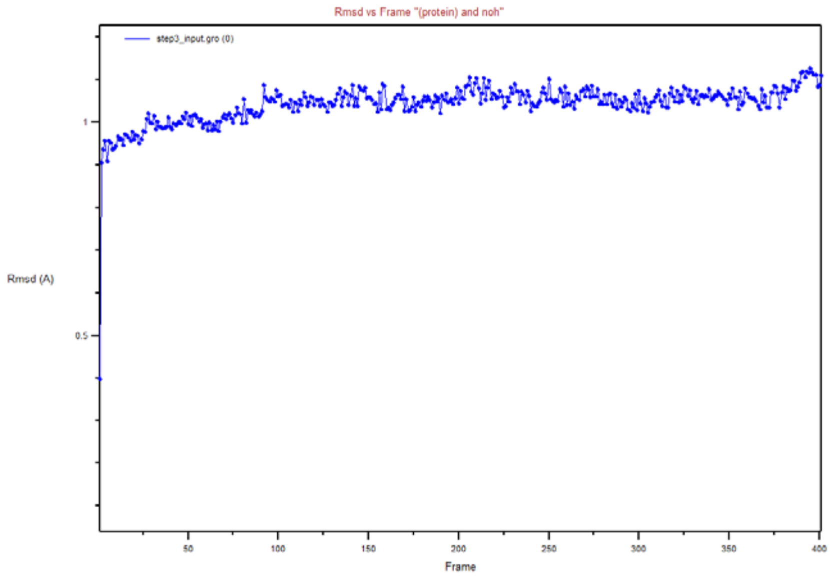

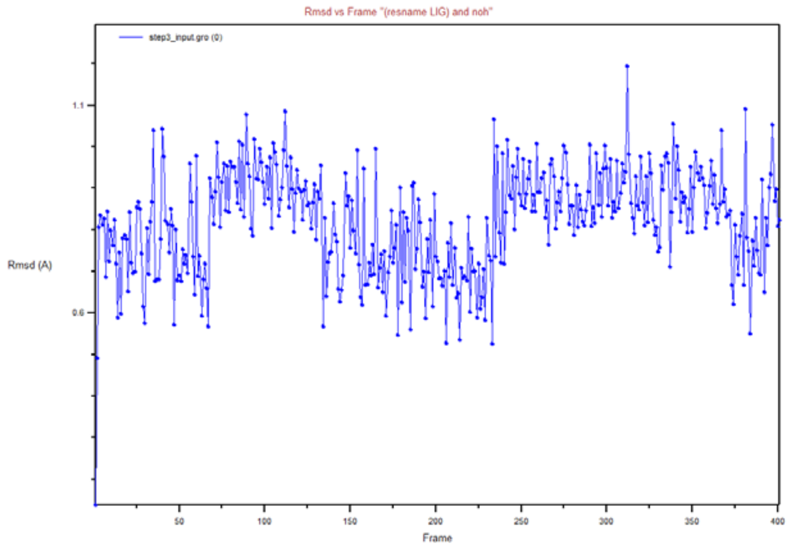

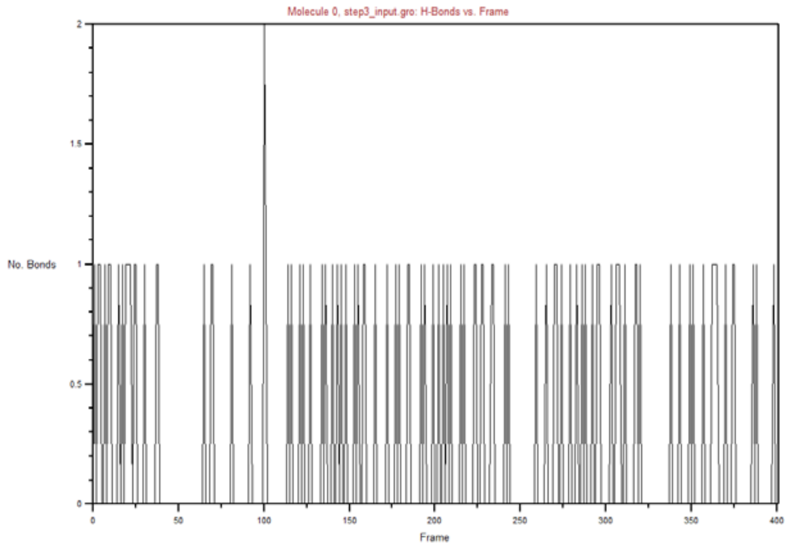

Supplementary Figure S3. Molecular dynamics simulation of 4S-C2_7α-GR complex during 4 ns. The stability of the complex was simulated using molecular dynamics in the GROMACS program

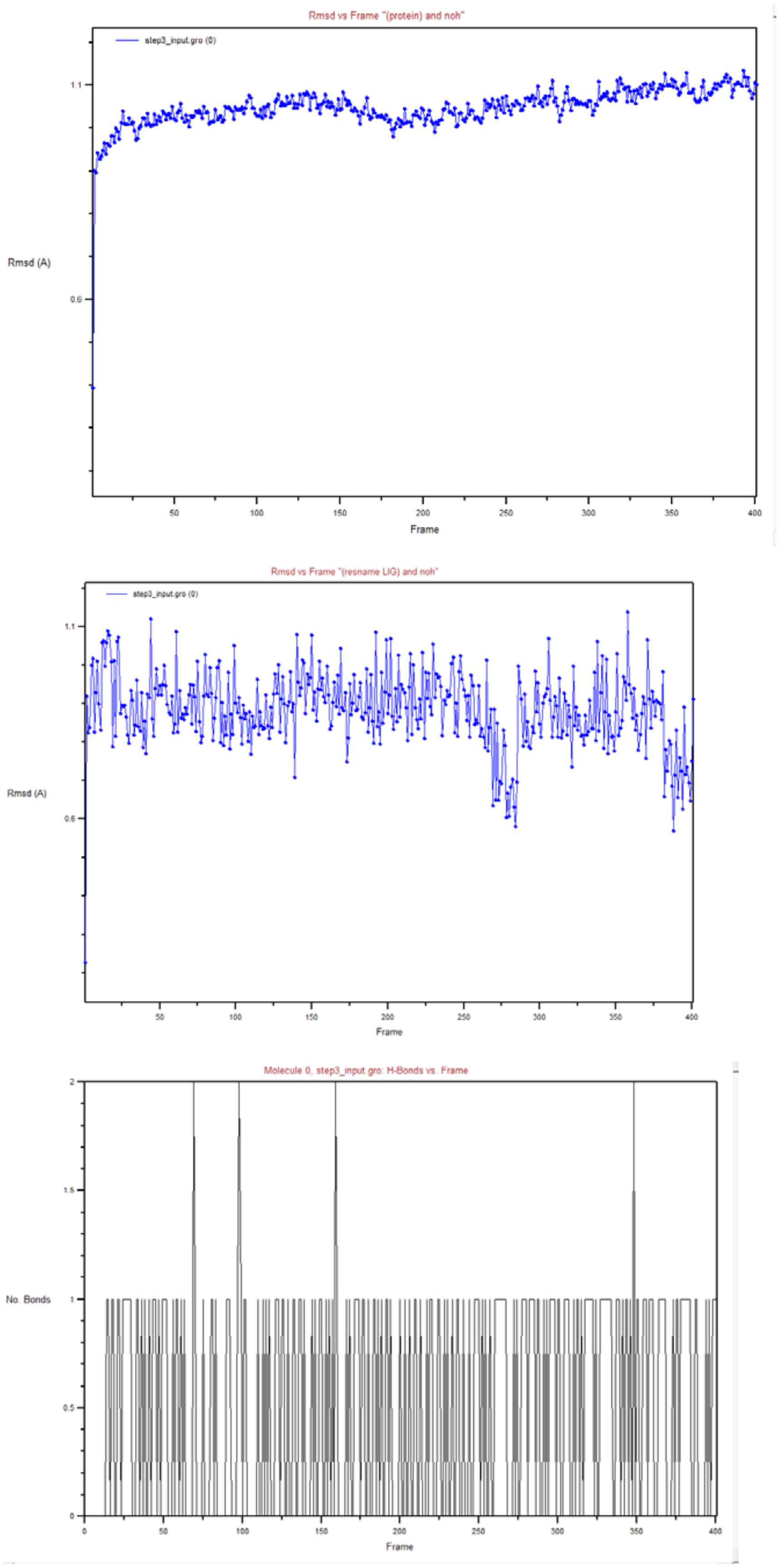

Supplementary Figure S4. Molecular dynamics simulation of 4S-C2_7β-GR complex during 4 ns. The stability of the complex was simulated using molecular dynamics in the GROMACS program

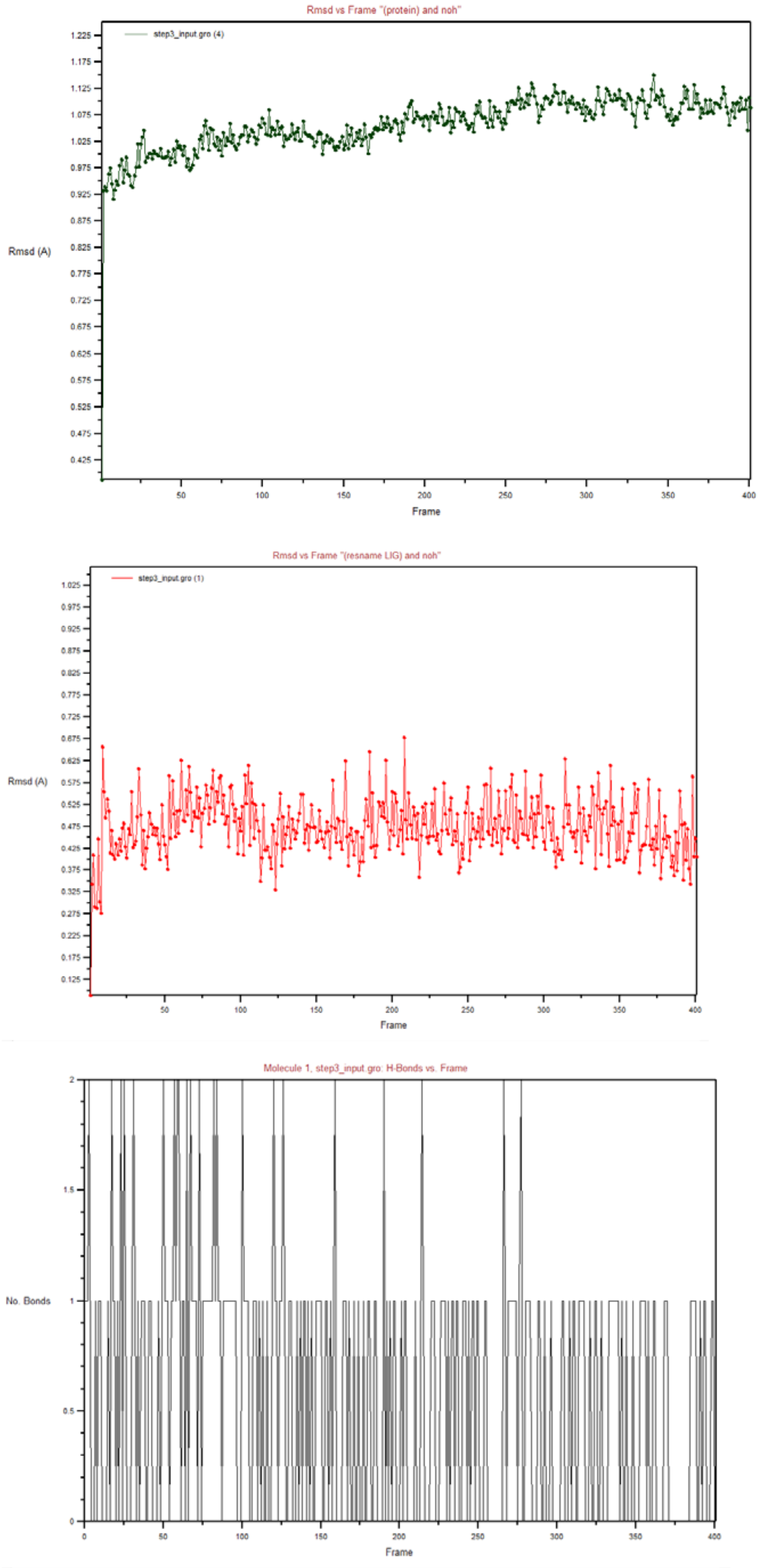

Supplementary Figure S5. Molecular dynamics simulation of 8S-E3_7α-GR complex during 4 ns. The stability of the complex was simulated using molecular dynamics in the GROMACS program

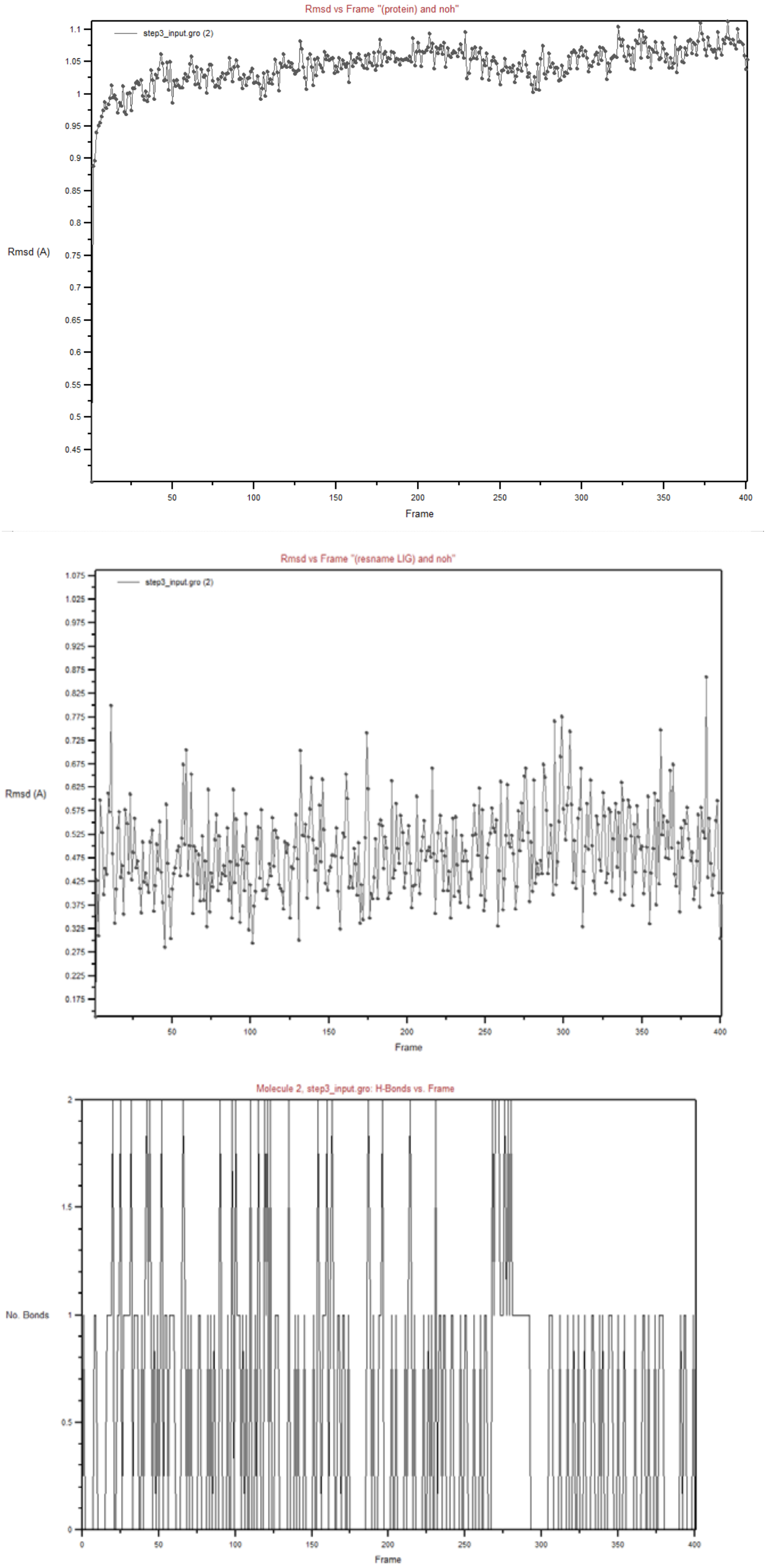

Supplementary Figure S6. Molecular dynamics simulation of 8S-E3_7β-GR complex during 4 ns. The stability of the complex was simulated using molecular dynamics in the GROMACS program

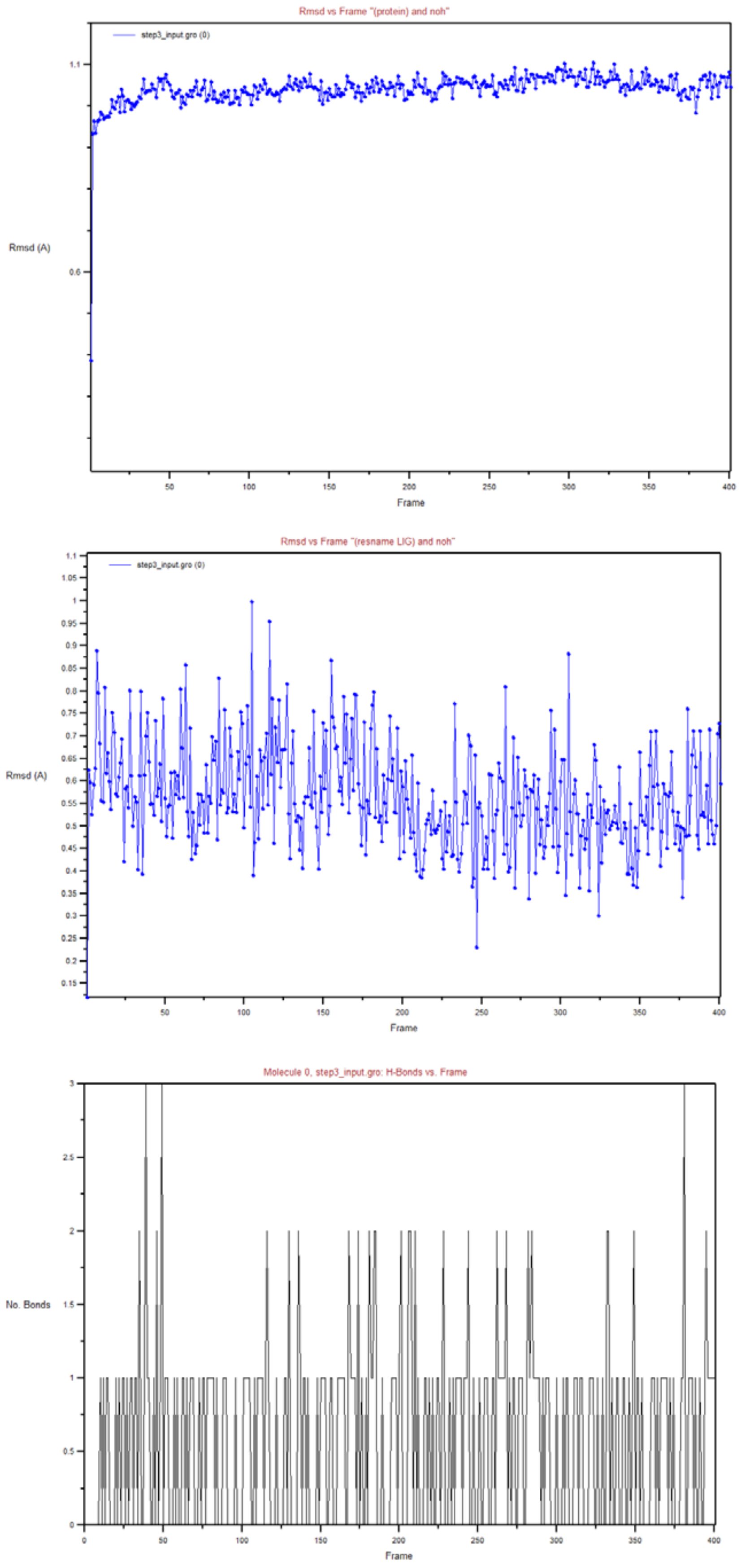

Supplementary Figure S7. Molecular dynamics simulation of 10S-E2_7α-GR complex during 4 ns. The stability of the complex was simulated using molecular dynamics in the GROMACS program

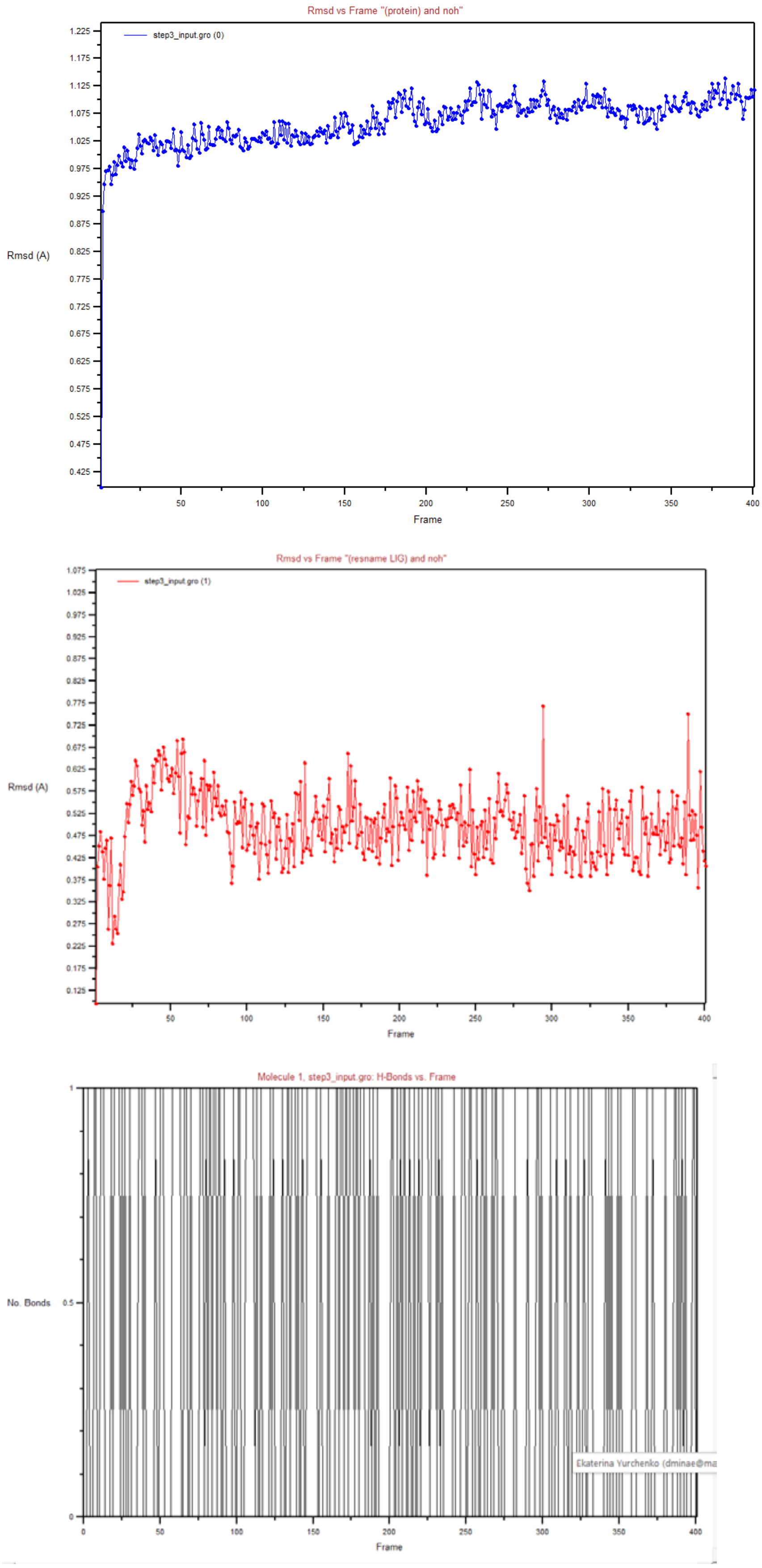

Supplementary Figure S8. Molecular dynamics simulation of 10S-E2_7β-GR complex during 4 ns. The stability of the complex was simulated using molecular dynamics in the GROMACS program

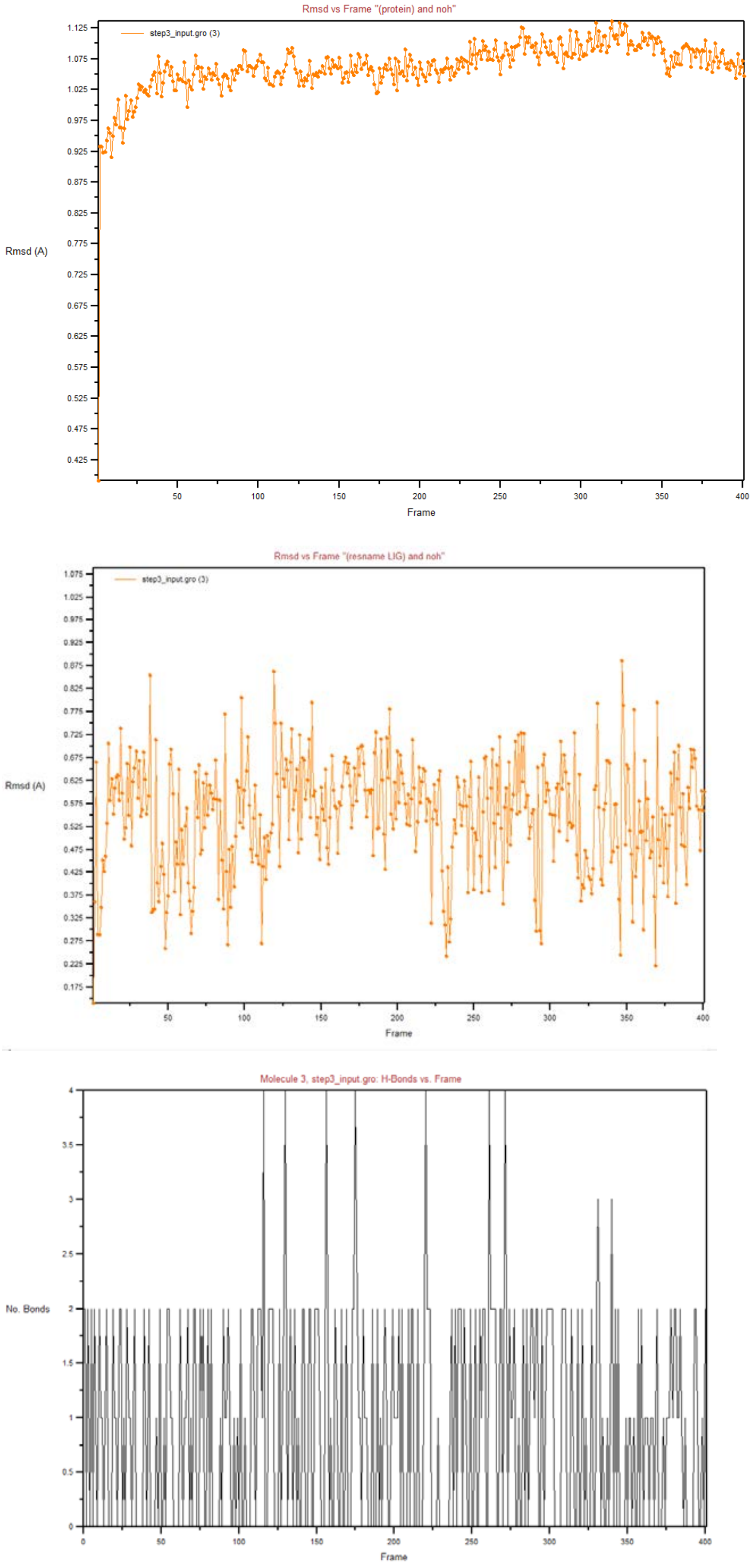

Supplementary Figure S9. Molecular dynamics simulation of 13S-G2_7α-GR complex during 4 ns. The stability of the complex was simulated using molecular dynamics in the GROMACS program

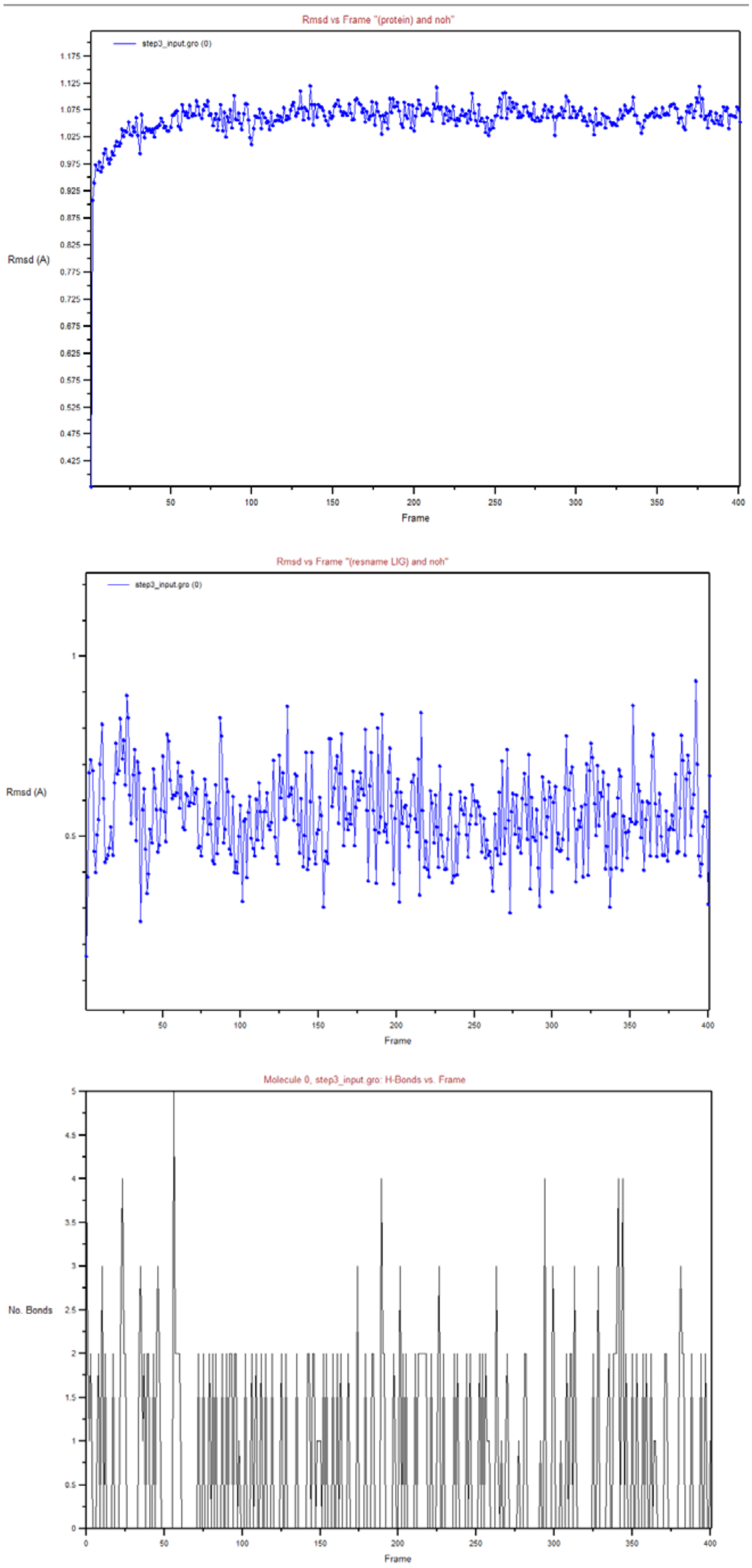

Supplementary Figure S10. Molecular dynamics simulation of 13S_G2_7β-GR complex during 4 ns. The stability of the complex was simulated using molecular dynamics in the GROMACS program

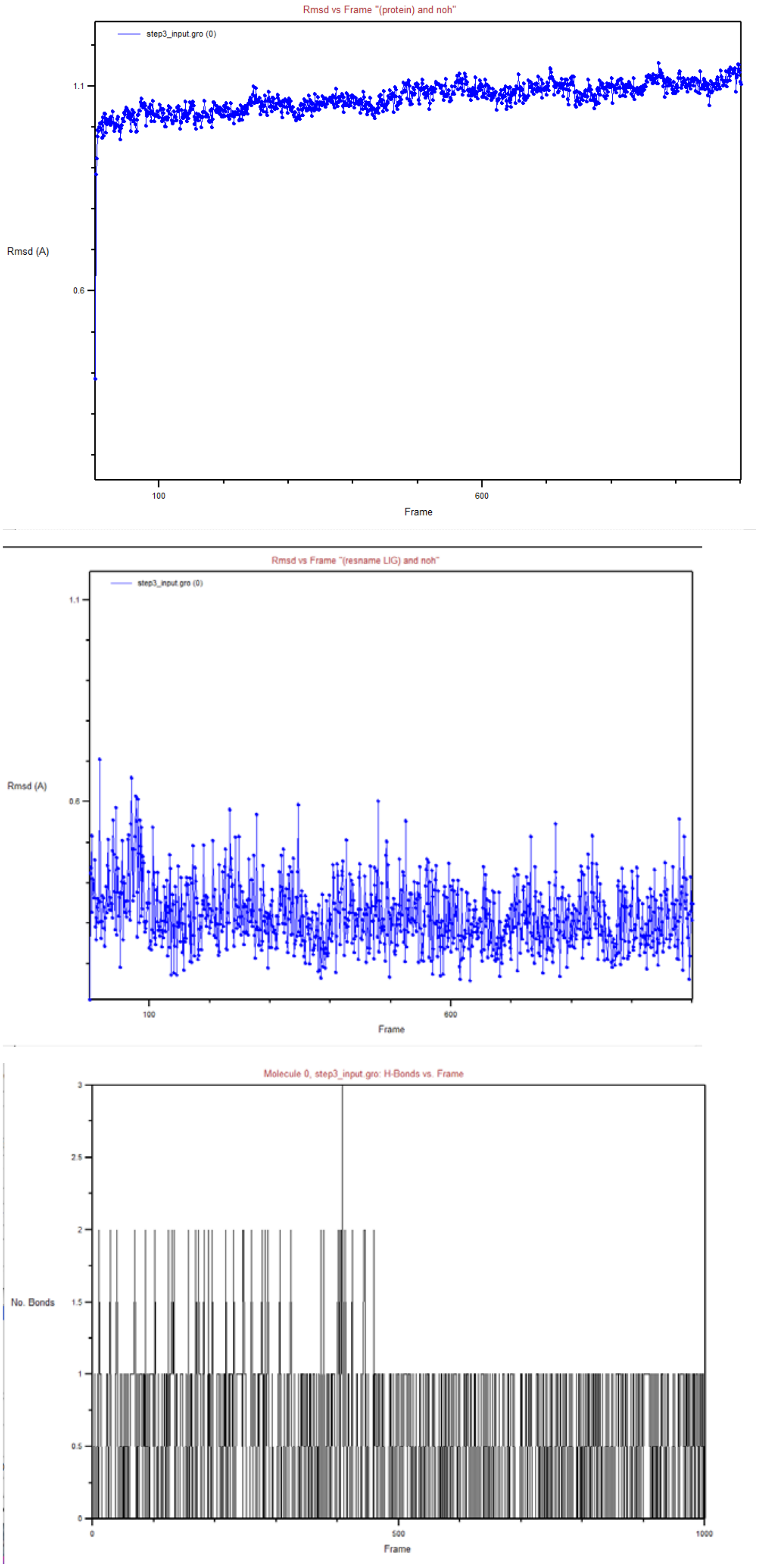

Supplementary Figure S11. Molecular dynamics simulation of 10S-E2_7α-GR complex during 10 ns. The stability of the complex was simulated using molecular dynamics in the GROMACS program

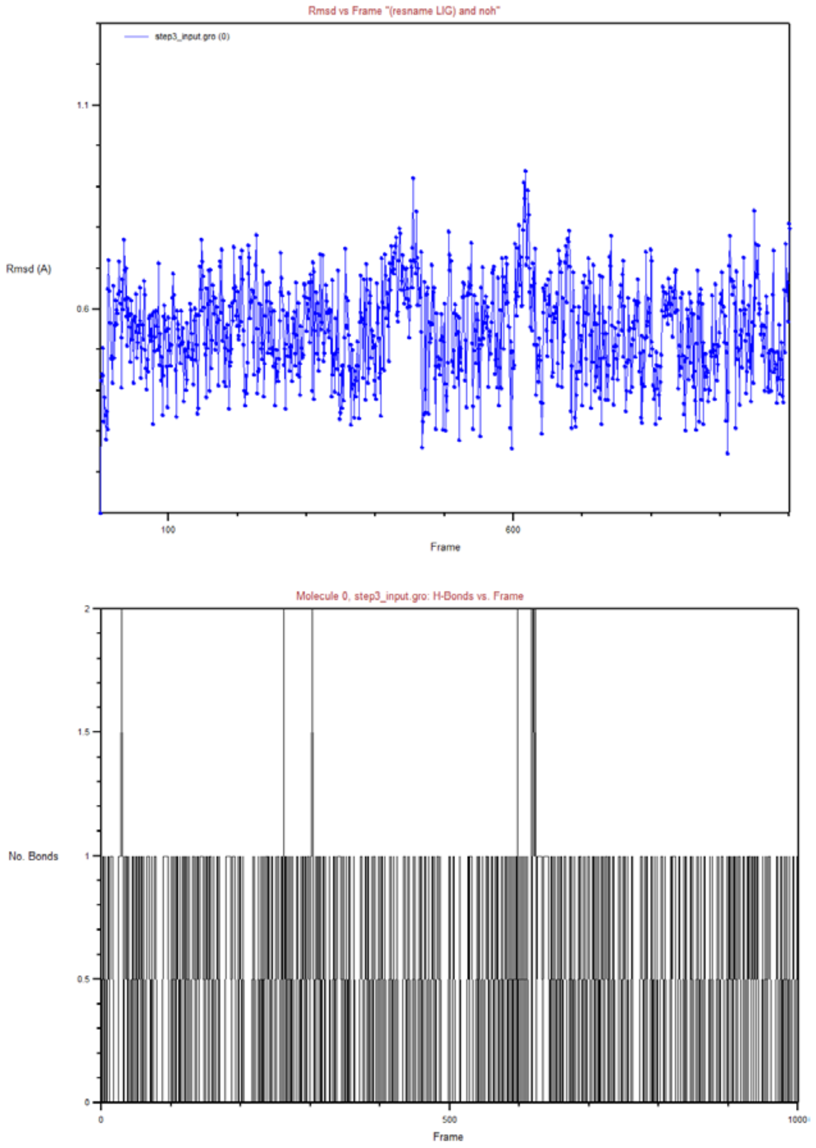

Supplementary Figure S12. Molecular dynamics simulation of 10S-E2_7β-GR complex during 10 ns. The stability of the complex was simulated using molecular dynamics in the GROMACS program

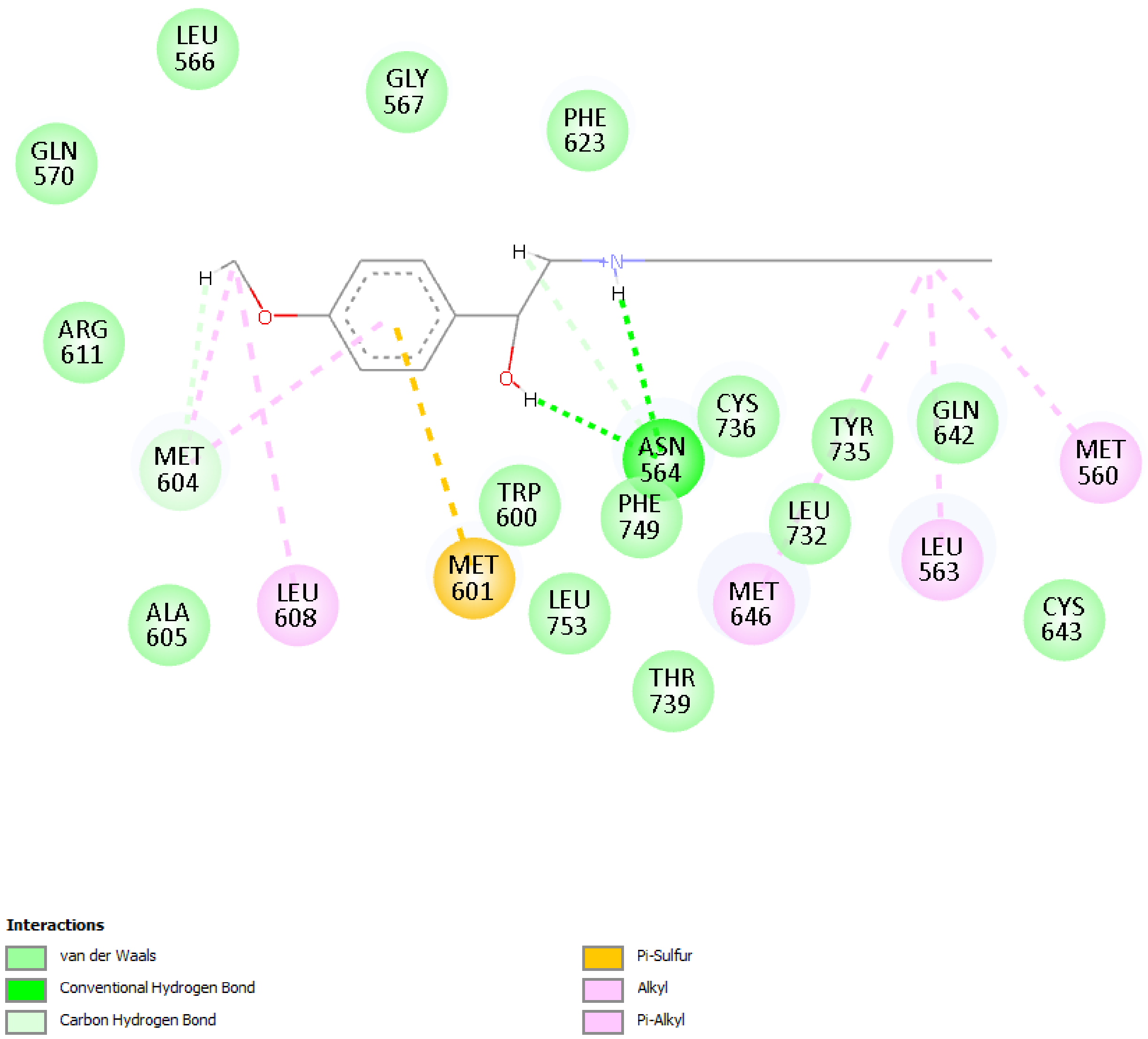

Supplementary Figure S13. 2D molecular docking diagram for 4S-C2_7α-GR interaction. 2D diagrams of ligand-protein interactions were obtained using BIOVIA Discovery Studio Visualiser v.25.1.0.24284

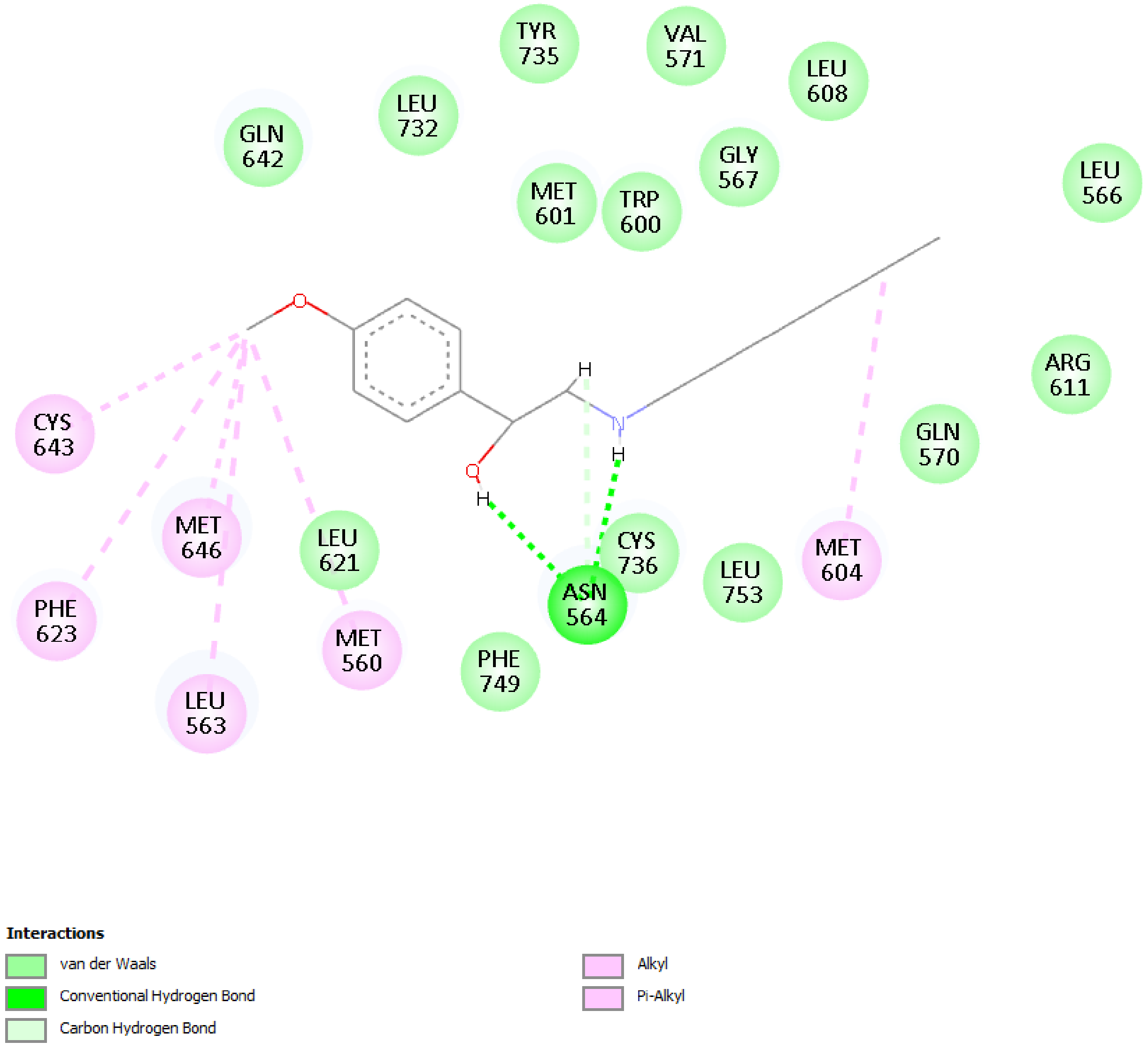

Supplementary Figure S14. 2D molecular docking diagram for 4S-C2_7β -GR interaction. 2D diagrams of ligand-protein interactions were obtained using BIOVIA Discovery Studio Visualiser v.25.1.0.24284

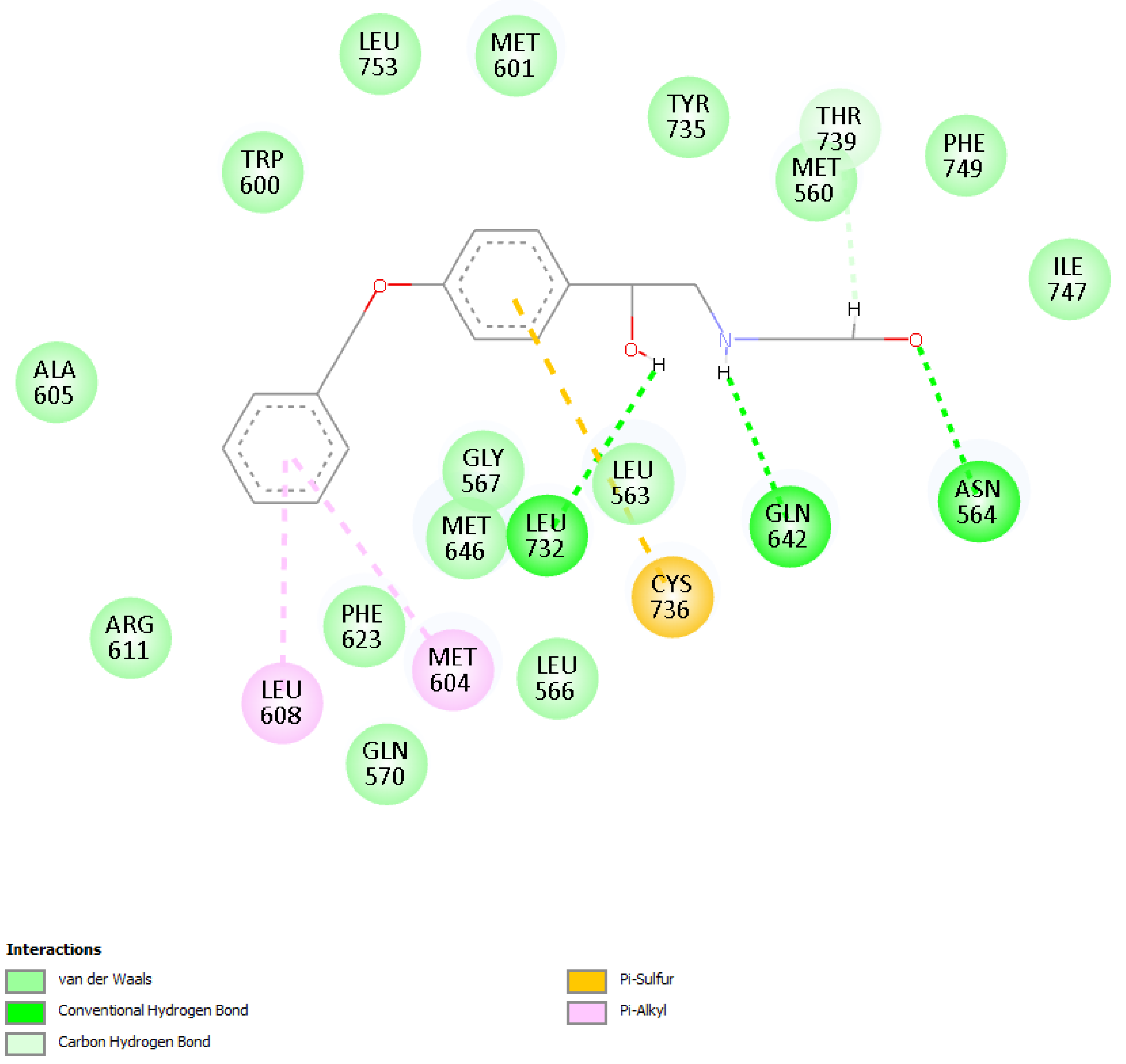

Supplementary Figure S15. 2D molecular docking diagram for 8S-E3_7α -GR interaction. 2D diagrams of ligand-protein interactions were obtained using BIOVIA Discovery Studio Visualiser v.25.1.0.24284

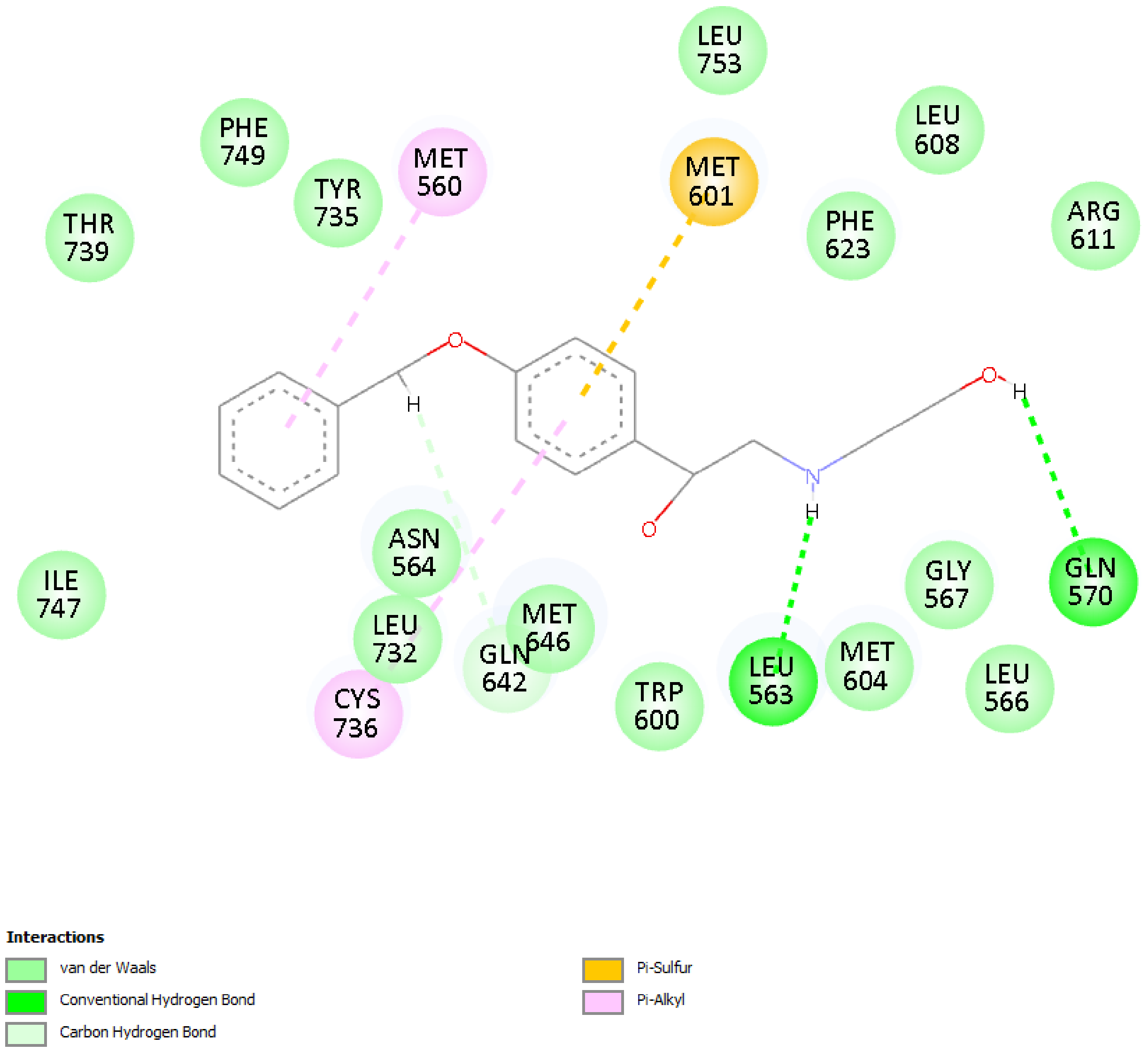

Supplementary Figure S16. 2D molecular docking diagram for 8S-E3_7β -GR interaction. 2D diagrams of ligand-protein interactions were obtained using BIOVIA Discovery Studio Visualiser v.25.1.0.24284

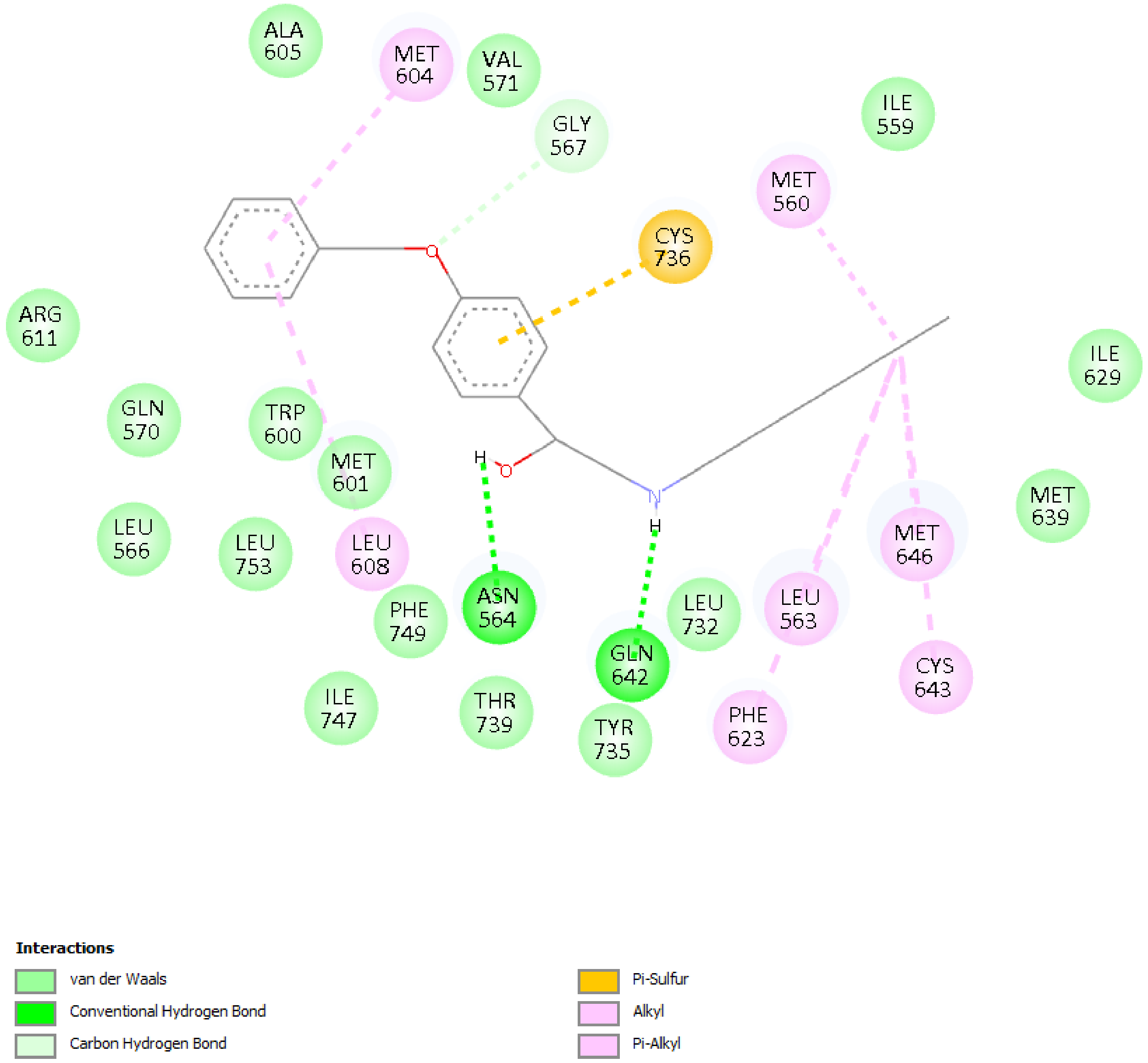

Supplementary Figure S17. 2D molecular docking diagram for 10S-E2_7α -GR interaction. 2D diagrams of ligand-protein interactions were obtained using BIOVIA Discovery Studio Visualiser v.25.1.0.24284

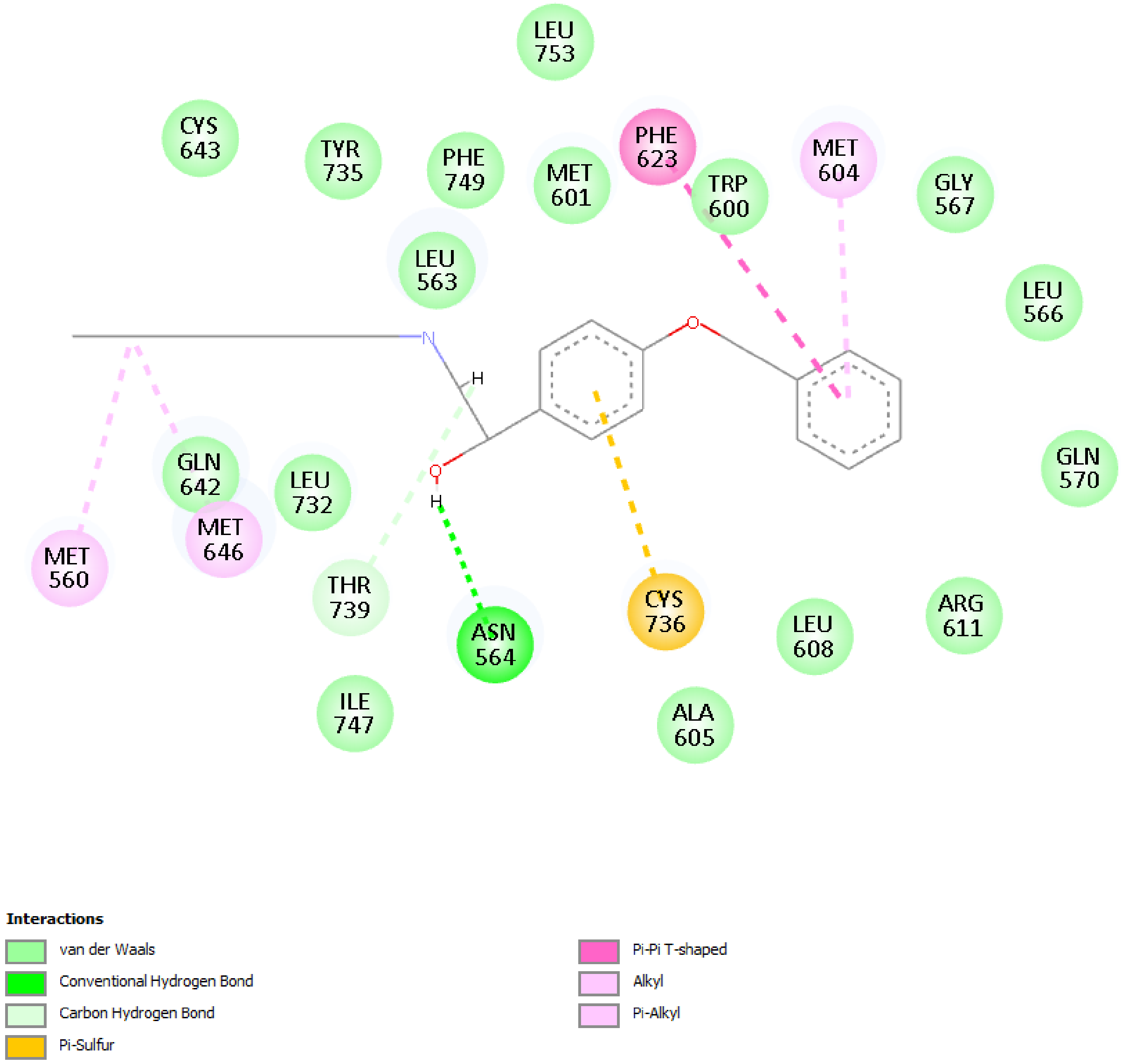

Supplementary Figure S18. 2D molecular docking diagram for 10S-E2_7β -GR interaction. 2D diagrams of ligand-protein interactions were obtained using BIOVIA Discovery Studio Visualiser v.25.1.0.24284

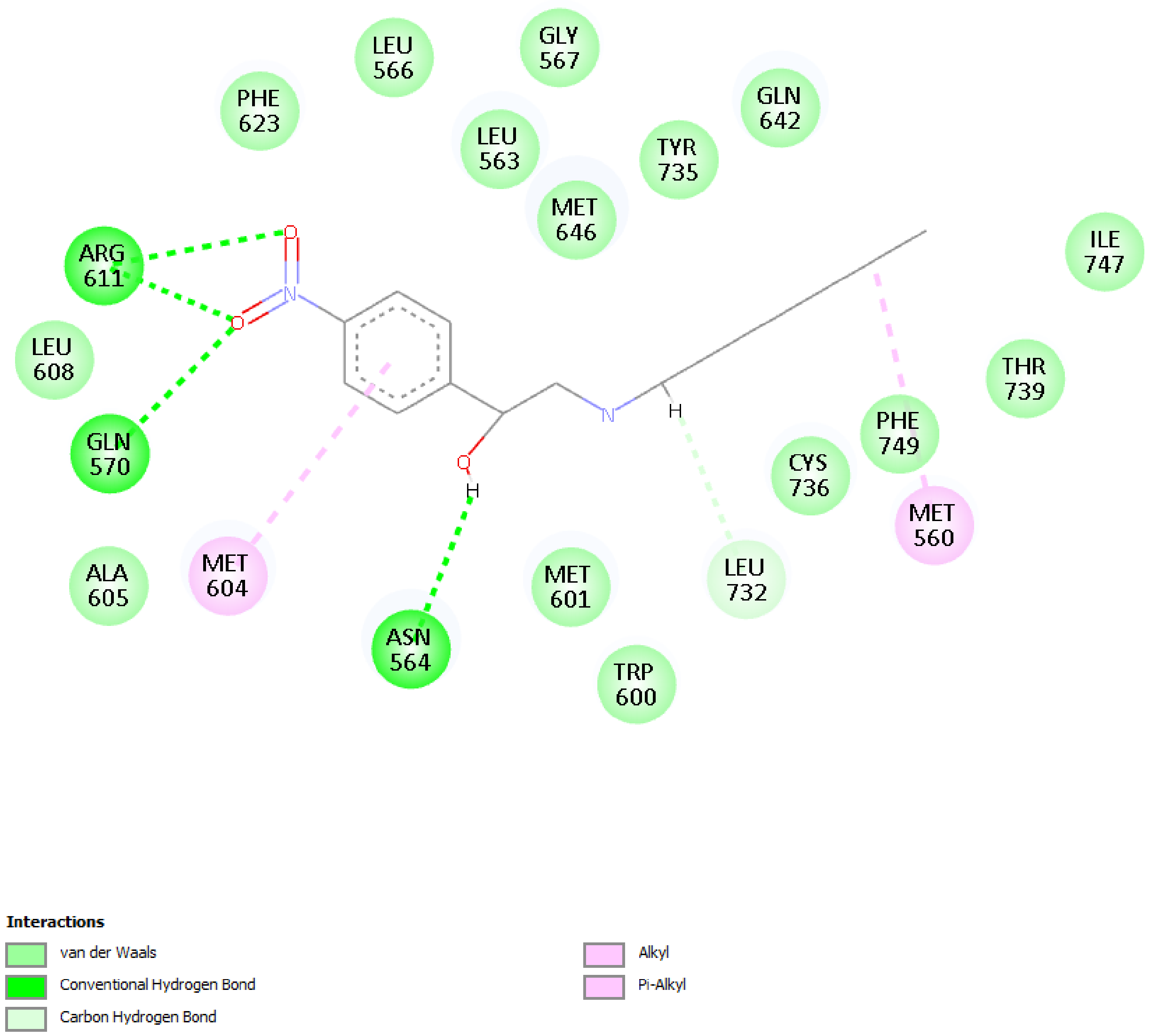

Supplementary Figure S19. 2D molecular docking diagram for 13S-G2_7α -GR interaction. 2D diagrams of ligand-protein interactions were obtained using BIOVIA Discovery Studio Visualiser v.25.1.0.24284

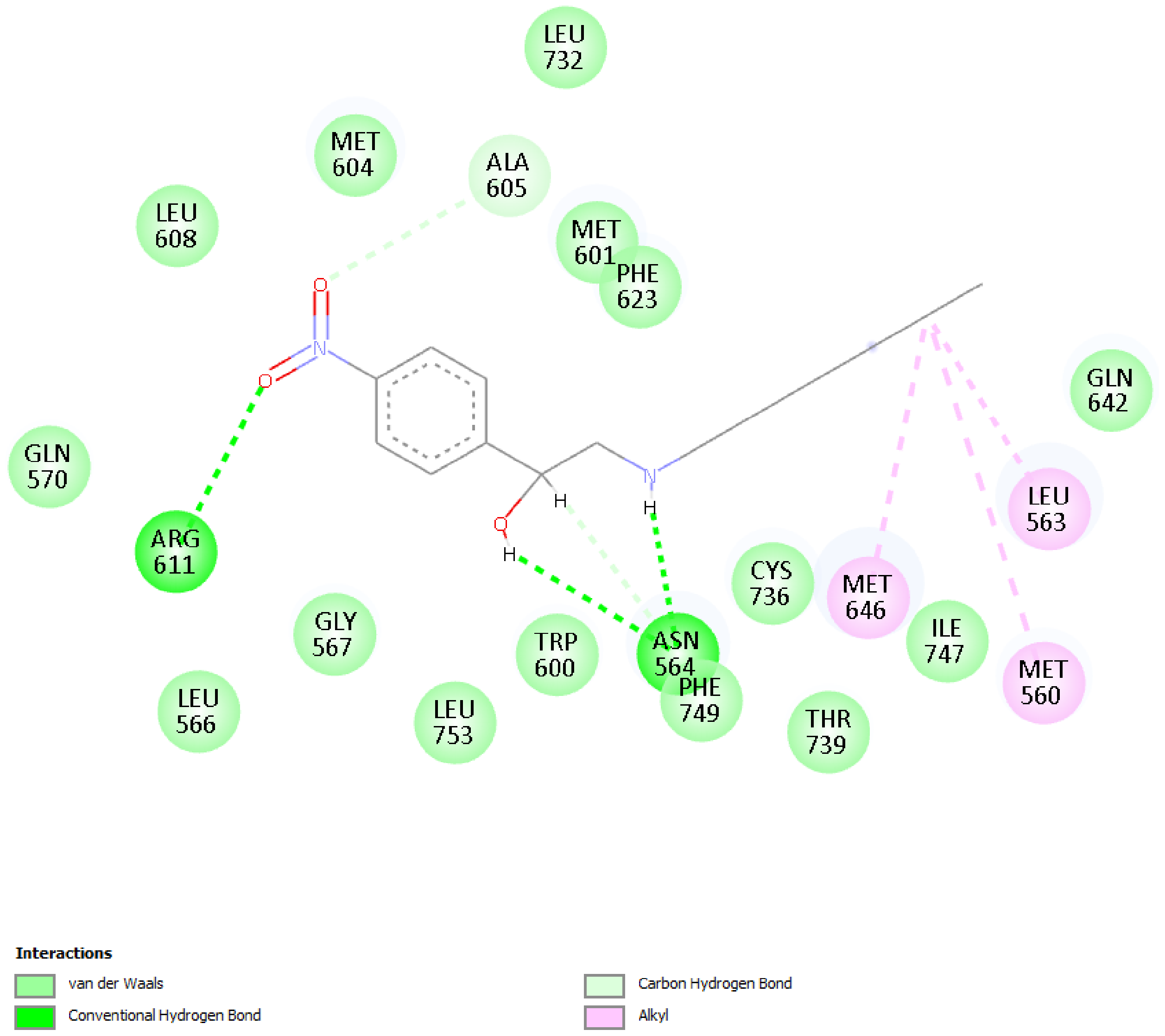

Supplementary Figure S20. 2D molecular docking diagram for 13S-G2_7β -GR interaction. 2D diagrams of ligand-protein interactions were obtained using BIOVIA Discovery Studio Visualiser v.25.1.0.24284

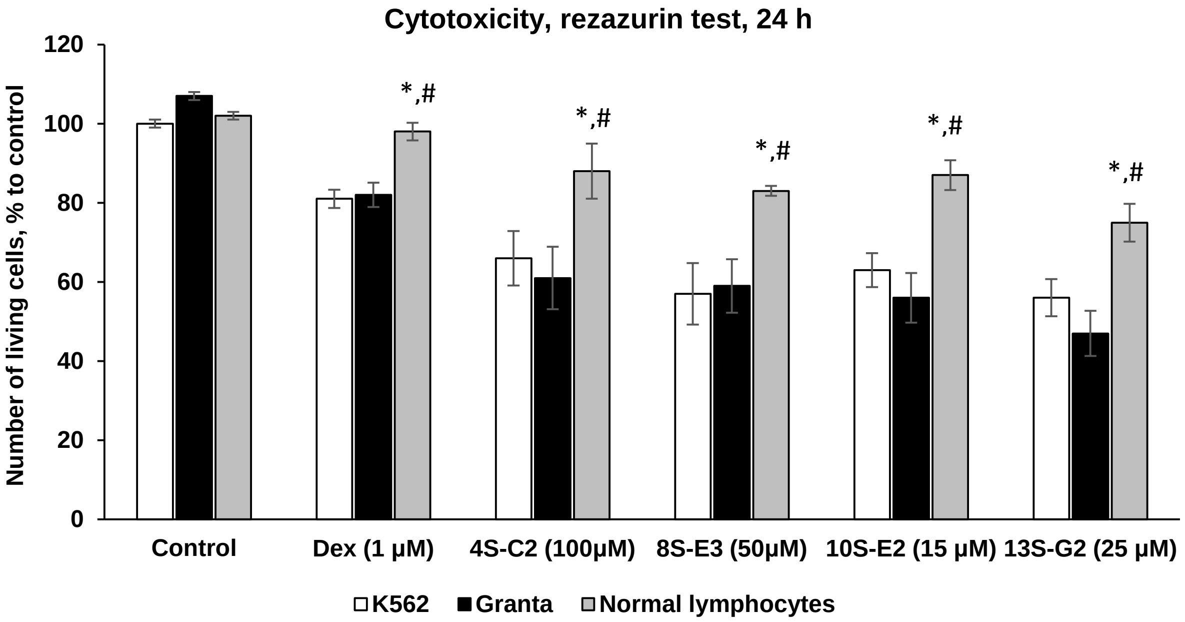

Supplementary Figure S21. Comparative cytotoxic effects of Dex and synephrine derivatives on K562 cells, Granta cells and normal human monocytes. K562 cells, Granta cells and normal human monocytes were treated with solvent (Control), Dex (1 μM), 4S-C2 (100 μM), 8S-E3 (50 μM), 10S-E2 (15 μM) and 13S-G2 (25 μM) for 24 h. Rezazurin assay was performed in triplets. Statistically significant differences as compared to: *-K562 cells; #-Granta cells ($p<0.05$) as and where reflected.

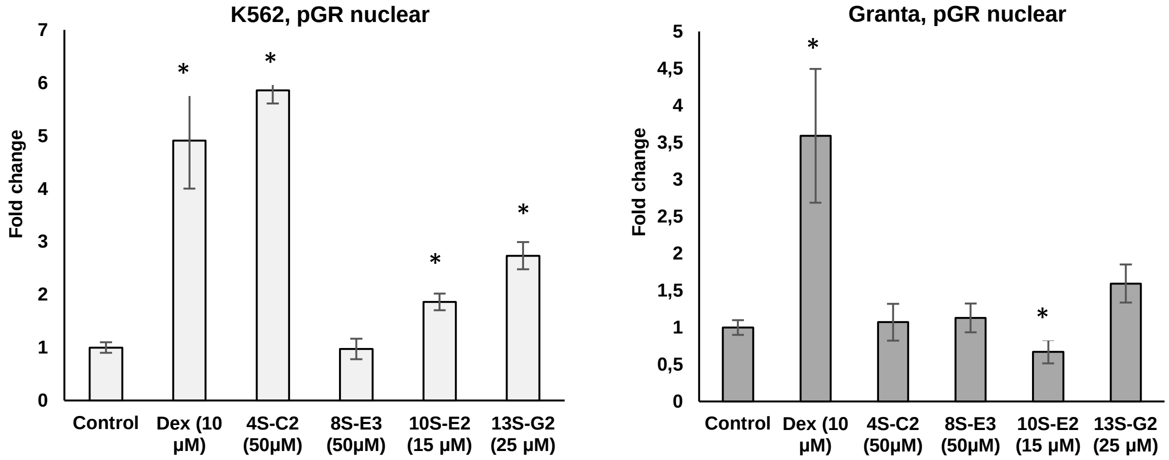

Supplementary Figure S22. Quantification of Western blot analysis. K562 and Granta cells were treated with solvent (Control), Dex (10 μM), 4S-C2 (50 μM), 8S-E3 (50 μM), 10S-E2 (15 μM) and 13S-G2 (25 μM) for 10 h. Quantification of Western blot analysis of p-GR protein levels was done using Image J free software. Statistically significant differences as compared to control: * - $p<0.05$

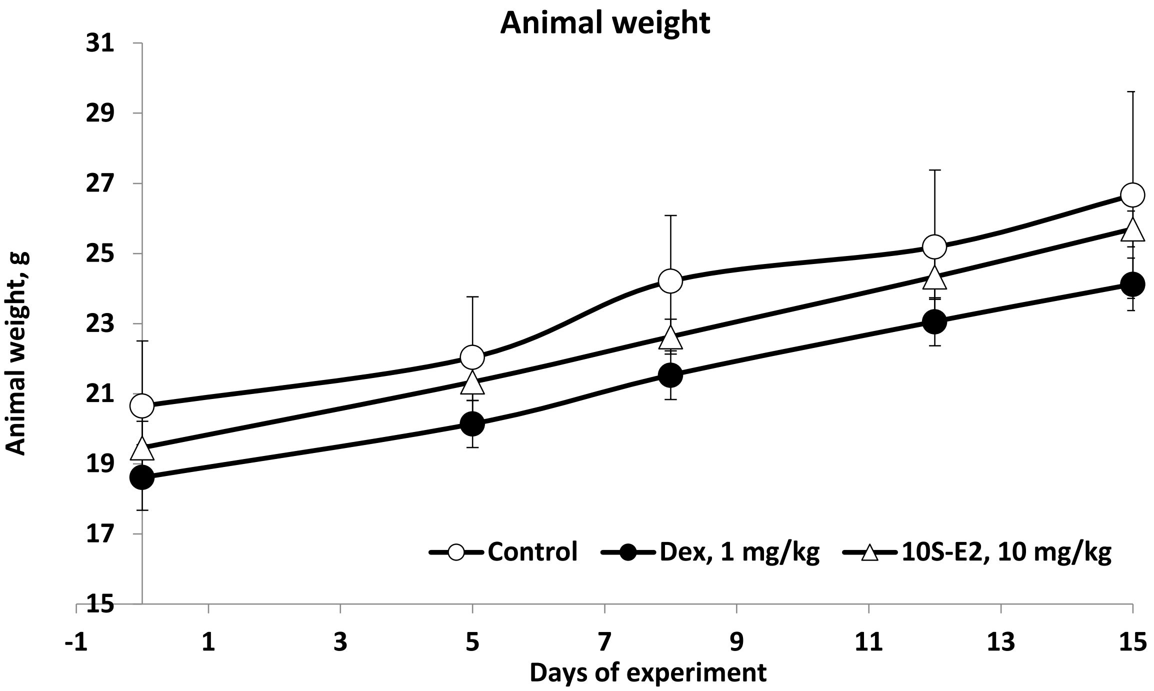

Supplementary Figure S23. Effect of 10S-E2 and Dex on animal body weight in the study of anti-cancer activity on P388 lymphoma model. *In vivo* anticancer activity was evaluated using the transplantable P388 murine lymphoma model. Animals were treated i.p. 3 times per week with Dex (1 mg/kg), 10S-E2 (10 mg/kg) or solvent (ethanol:tween 80:distilled water=0.5:0.5:9). Body weight was measured twice a week. There were no statistically significant differences in body weight changes between experimental and control groups

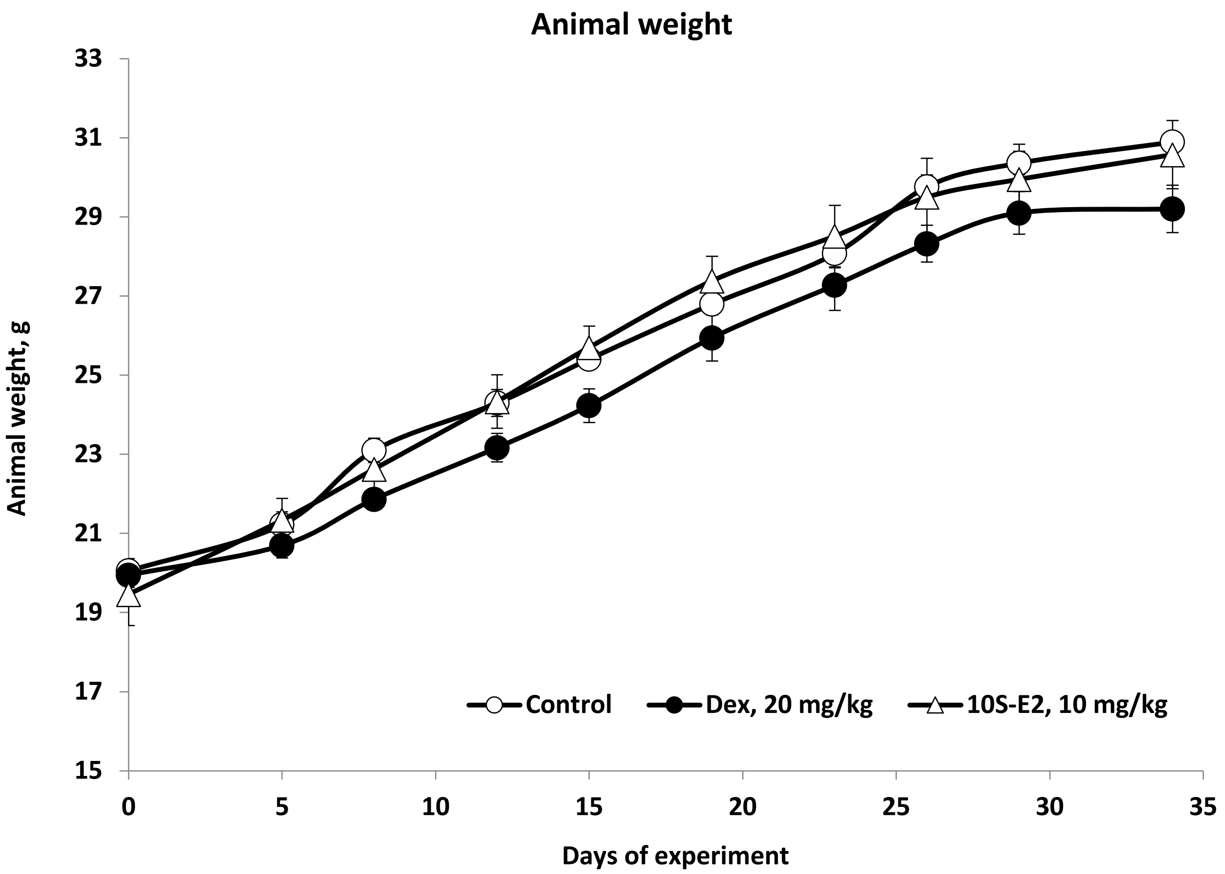

Supplementary Figure S24. Effect of 10S-E2 and Dex on animal body weight in glucocorticoid-induced osteoporosis study. GIOP was induced by i.p. injection of Dex (20 mg/kg) or 10S-E2 (10 mg/kg) in 12 weeks old BALB/c female mice every 24 h for 5 weeks. Body weight was measured twice a week. There were no statistically significant differences in body weight changes between experimental and control groups
